# One-Pot Iridium Catalyzed C–H Borylation/Sonogashira Cross-Coupling: Access to Borylated Aryl Alkynes

**DOI:** 10.3390/molecules25071754

**Published:** 2020-04-10

**Authors:** Ghayoor A. Chotana, Jose R. Montero Bastidas, Susanne L. Miller, Milton R. Smith, Robert E. Maleczka

**Affiliations:** 1 Department of Chemistry, Michigan State University, East Lansing, MI 48824-1322, USA; ghayoor.abbas@lums.edu.pk (G.A.C.); monter20@chemistry.msu.edu (J.R.M.B.); 2 Department of Chemistry and Chemical Engineering, Syed Babar Ali School of Science & Engineering, (SBASSE), Lahore University of Management Sciences (LUMS), Sector U, DHA, Lahore Cantt. 54792, Pakistan; 3 BoroPharm Inc., 39555 Orchard Hill Place, Suite 600, Novi, MI 48375, USA; mille262@chemistry.msu.edu

**Keywords:** C–H borylation, Sonogashira cross-coupling, borylated aryl alkynes, one-pot reaction

## Abstract

Borylated aryl alkynes have been synthesized via one-pot iridium catalyzed C–H borylation (CHB)/Sonogashira cross-coupling of aryl bromides. Direct borylation of aryl alkynes encountered problems related to the reactivity of the alkyne under CHB conditions. However, tolerance of aryl bromides to CHB made possible a subsequent Sonogashira cross-coupling to access the desired borylated aryl alkynes.

## 1. Introduction

Boronic acids and esters serve as precursors for a variety of functional groups and as synthetic handles for C–C bond formation [1,2]. Over the past two decades, iridium-catalyzed C–H borylation (CHB) of arenes have emerged as useful additions to the synthetic chemist’s toolbox [3,4,5,6,7]. The regiochemistry of iridium-catalyzed CHB of arenes is traditionally governed by sterics [3,7,8]; often complementing regiochemical outcomes of electrophilic aromatic substitution and directed *ortho* metalation. Since its discovery [9], methods to expand regiocontrol (*ortho*, *meta,* and *para*) [10,11,12], sp^3^ borylation protocols [13,14,15,16,17,18,19,20,21] and one-pot reactions [22,23,24,25,26,27] have been developed. 

**Scheme 1:**
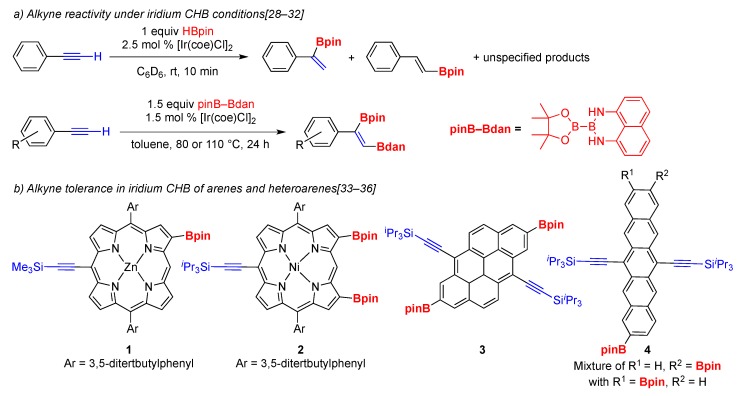
(**a**) Reactivity and (**b**) tolerance of alkynes in iridium C–H borylations [28–36}.

In contrast, few tactical advances have expanded the chemoselectivity of iridium-catalyzed CHBs. This is not to say that CHBs have poor functional group tolerance. Ester, amide, ether, carbamate, and nitrile functionalities are all well tolerated. Satisfactorily, CHB of halogenated arenes leaves the carbon-halogen bonds intact, which differs from other protocols involving palladium or nickel. In contrast, substrates bearing alkenes or unhindered alkynes have been considered problematic owing to the propensity of these groups to react under the borylation conditions. In fact, addition of hydroborane or diboron reagents across triple bonds can occur with catalytic systems similar to the traditional conditions used for CHB (Scheme 1a) [28,29,30,31,32]. However, there are reports in which CHB of arenes or heteroarenes bearing an alkyne functionality have been successful (Scheme 1b) [33,34,35,36]. It is likely that in these examples the presence of two bulky substituents on the alkyne hinder its reactivity, allowing for chemoselective borylation of the porphyrin moiety (**1**, **2**) or the polyarene skeleton (**3**, **4**). The dichotomy of these results was the first subject of our study.

Unwanted alkyne reactivity can be viewed as a CHB limitation, since borylated aromatic alkynes have found use in the synthesis of extensively conjugated polymeric materials [37] and in crystal engineering, biological inhibition, molecular sensing, chirality, and structural assignment, etc., [38,39,40,41]. The preparation of borylated aromatic alkynes usually involves introduction of the boronic ester/acid functionality on an aromatic alkyne by metalation/borylation [42,43] or Pd-catalyzed borylation of aromatic halides [44]. We hypothesized that by courtesy of CHB halogen tolerance it would be possible to make such intermediates in the opposite order, namely, to synthesize borylated aromatic alkynes by a CHB/Sonogashira coupling sequence. If such a sequence could also be accomplished in a one-pot fashion, it would streamline the synthesis of borylated aromatic alkynes while allowing access to target molecules bearing the contra-electronic substitution patterns often associated with CHB reactions. 

## 2. Results and Discussion

**Scheme 2:**
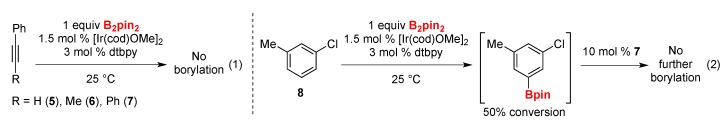
Attempted CHB in the presence of alkynes.

The prior art was inconclusive as to the compatibility between alkynes and CHB conditions. Therefore, we began by subjecting alkynyl arenes to CHB conditions (Scheme 2, Equation (1)). Attempted borylation of phenyl acetylene (**5**) using the [Ir(cod)OMe]_2_/dtbpy catalyst system was unsuccessful. Considering that the terminal C–H bond in acetylene may be too acidic, we examined the borylation of 1-phenyl-1-propylene (**6**) and diphenyl acetylene (**7**). Neither of these alkynes underwent aromatic borylation. It was also found that the addition of 10 mol % of diphenylacetylene (**7**) halts the ongoing borylation of an otherwise suitable CHB substrate as shown in Scheme 2, Equation (2). Furthermore, attempted borylation of diphenyl acetylene with an (Ind)Ir(cod)/ dmpe catalyst system at 150 °C gave a mixture of products arising from hydrogenation, hydroboration, and catalytic borylation. These results suggest that the alkynyl group binds tightly to the active borylation catalyst at 25 °C, but at elevated temperatures the alkynyl group becomes a reactive partner.

**Scheme 3:**
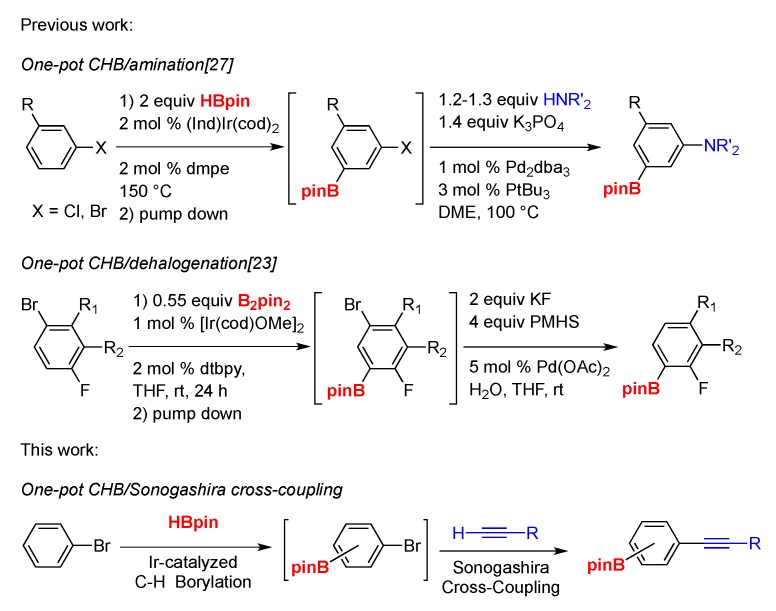
One-pot CHB of aryl halides followed by selective reaction of the C–Halogen bond [23,27].

These results drove our decision to develop a CHB/Sonogashira protocol. Others had demonstrated the tolerance of boronic esters under Sonogashira cross-coupling reaction conditions [39,40,45,46,47]. While our group previously showed that despite the propensity for self-Suzuki reactions, one-pot reactions involving CHB of aryl halides followed by C–N cross-coupling of the C–halogen bond [27] or dehalogenation [23], that keep the C–B bond intact are possible (Scheme 3). These studies provided the foundation from which we would seek to establish a one-pot CHB/Sonogashira cross-coupling of aryl halides to access borylated aryl alkynes.

**Scheme 4:**
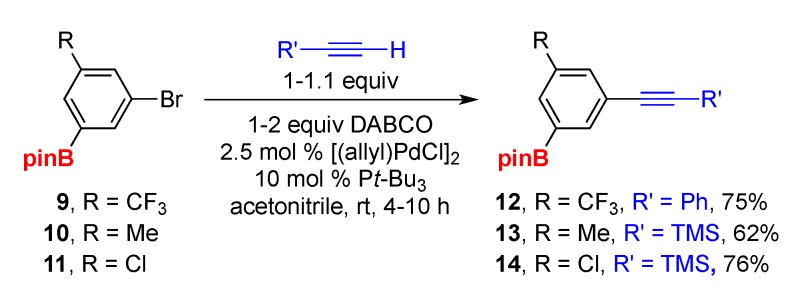
Sonogashira cross-coupling of an aryl bromide boronic ester.

3-Bromobenzotrifluoride was chosen as our test substrate. First, borylated 3-bromobenzotrifluoride (**9**) was subjected to Sonogashira cross-coupling under Fu’s conditions using phenyl acetylene (**5**) and CuI cocatalyst [48]. We were pleased to observe the formation of the desired borylated aromatic alkyne without any significant deborylation or polyphenylene formation. However, the reaction had stopped at about 90% conversion after 18 h and homocoupling of the alkyne was observed by GC-MS. As Buchwald had shown that a copper co-catalyst may inhibit Sonogashira coupling [49] and given that CuI can promote oxidative homocoupling of alkynes, we shifted to copper-free conditions reported by Soheili [50]. This resulted in full conversion of substrate in 10 h and the resulting borylated aromatic alkyne was isolated in 75% yield (Scheme 4). We used this protocol with a couple of other aryl borylated bromides (**10**, **11**) and the Sonogashira products were obtained in good yields (**13**, **14**). Synthesis of **14** was run in a bigger scale (10 g, 31.5 mmol) which shows the robustness of this reaction.

With this success, we moved on to developing the one-pot borylation/Sonogashira sequence. In addition to polyphenylene formation and deborylation, we envisioned other potential issues negatively impacting this approach, such as residual iridium catalyst/ligand affecting the subsequent Sonogashira coupling. Iridium is also known to catalyze the polymerization of aromatic alkynes [51]. In practice, 3-bromobenzotrifluoride was borylated using a (Ind)Ir(cod)/dmpe catalyst system and the intermediate boronate ester was then subjected to Sonogashira coupling without isolation. The coupling went smoothly without any interference from residual iridium catalyst, ligand, or borylation by-products and the desired product was isolated in 64% yield (Table 1, entry 1). Other substrates reacted similarly with phenyl acetylene or TMS acetylene as the alkyne partner. The general one-pot borylation/Sonogashira coupling sequence and the product yields over two-steps are presented in Table 1.

**Scheme 5:**
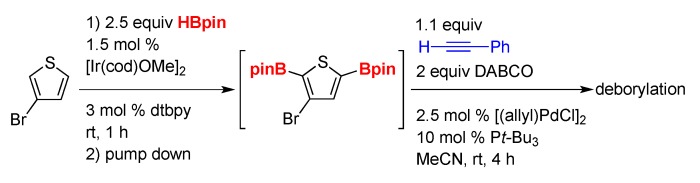
Attempted di-borylation Sonogashira cross-coupling of 3-bromothiophene.

Both electron rich as well as electron deficient aryl bromides proved to be efficient substrates. Entries 10 and 11 show that a hindered C–Br bond in a bromoaryl boronate ester can undergo selective Sonogashira coupling without any deborylation of the more sterically accessible C–B bond. Double Sonogashira coupling can be carried out starting from 1,3-dibromobenzene (entry 12). Attempted mono-Sonogashira coupling on the intermediate boronic ester of 1,2-di-bromobenzene using 0.9 equiv of TMS-acetylene resulted in a 1:3 mixture of two regioisomers, however the di-Sonogashira product was the major species observed by GC-FID. The resulting borylated aromatic enediynes were isolated in good yields by using 2.2 equiv of alkyne (entries 13 and 14). To expand the scope of this methodology to heteroaromatics, we examined the one-pot borylation/Sonogashira coupling of 3-bromothiophene. Diborylation was complete in 1 h, however upon exposure to the Sonogashira conditions, extensive deborylation was observed (Scheme 5).

Considering that the presence of iridium may have caused deborylation [52,53], we ran the Sonogashira coupling on isolated 2-bromo-5-Bpin-thiophene. Although the Sonogashira coupling was complete in 2 h, about 80% of the coupled product was deborylated. These results suggest that the presence of Bpin functionality on the 2-position of thiophene is inherently unstable to the Sonogashira conditions. Indeed, Zheng also reported deborylation during microwave-assisted Sonogashira coupling of 2-borylated heteroaromatics [46].

## 3. Materials and Methods 

### 3.1. Materials

All commercially available chemicals were used as received or purified as described. Bis(η^4^-1,5-cyclooctadiene)-di-*µ*-methoxy-diiridium(I) [Ir(cod)OMe]_2_ [54], (η^5^-Indenyl)(cyclooctadiene) iridium (Ind)Ir(cod)} [55], and pinacolborane (HBpin) [56] were prepared as per the literature procedures. 4′-Di-t-butyl-2,2′-bipyridine (dtbpy), bis(pinacolato)diboron (B_2_pin_2_), and 3-bromobenzonitrile were sublimed before use. Liquid aryl bromides were refluxed over CaH_2_, distilled, and degassed. Phenyl acetylene was distilled before use. Acetonitrile was distilled over activated molecular sieves. n-Hexane was refluxed over sodium, distilled, and degassed. Silica gel (230–400 Mesh) was purchased from EMD™.

### 3.2. General Procedure A: Sonogashira Cross-Coupling of Borylated Aryl Bromides

In a glove box, borylated aryl bromide (1.0 mmol, 1 equiv), 1,4-diazabicyclo[2.2.2]octane [DABCO] (225 mg, 2.0 mmol, 2 equiv), allylpalladium chloride dimer (9 mg, 0.025 mmol, 2.5 mol %), P*t-*Bu_3_ (20 mg, 0.1 mmol, 10 mol %), alkyne (1.1 mmol, 1.1 equiv), and acetonitrile (3 mL) were transferred into a Schlenk flask equipped with a magnetic stirring bar [50]. The flask was then stoppered and stirred at room temperature until the Sonogashira coupling was judged complete by GC-FID. After completion, 5 mL of water were added to the reaction mixture. The reaction mixture was extracted with ether (10 mL × 3). The combined ether extractions were washed with brine (10 mL), followed by water (10 mL), dried over MgSO_4_ before being concentrated under reduced pressure on a rotary evaporator. The crude material was then subjected to column chromatography.

### 3.3. General Procedure B: One-Pot CHB/Sonogashira Reaction

In a glove box, (Ind)Ir(cod) (8 mg, 0.02 mmol, 2 mol % Ir), dmpe (3 mg, 0.02 mmol, 2 mol %), HBpin (256 mg, 2.0 mmol, 2 equiv), and aryl bromide (1.0 mmol, 1 equiv) were transferred into a Schlenk flask equipped with a magnetic stirring bar. The flask was stoppered, removed from the glove box, and stirred at 150 °C until the borylation was judged completely by GC-FID/MS. The reaction mixture was allowed to cool to room temperature and subsequently placed under high vacuum for 1–2 h. The Schlenk flask was brought into the dry box and 1,4-diazabicyclo[2.2.2]octane [DABCO] (225 mg, 2.0 mmol, 2 equiv), allylpalladium chloride dimer (9 mg, 0.025 mmol, 2.5 mol %), P*t-*Bu_3_ (20 mg, 0.1 mmol, 10 mol %), alkyne (1.1–1.3 mmol, 1.1–1.3 equiv) and acetonitrile (3 mL) were added [50]. The flask was then stoppered and stirred at room temperature until the Sonogashira coupling was judged completely by GC-FID. After completion, 10 mL of water were added to the reaction mixture. The reaction mixture was extracted with ether (10 mL × 3). The combined ether extractions were washed with brine (10 mL), followed by water (10 mL), dried over MgSO_4_ before being concentrated under reduced pressure on a rotary evaporator. The crude material was then subjected to column chromatography.

### 3.4. Analytical data of products 12–25

*3-(Phenylethynyl)-5-(4,4,5,5-tetramethyl-1,3,2-dioxaborolane-2-yl)-benzotrifluoride (**12**)*

From Sonogashira coupling of borylated aryl bromide: the general procedure A was applied to the borylated version of 3-bromobenzotrifluoride (**9**, 351 mg, 1.0 mmol, 1 equiv) with phenyl acetylene (121 μL, 112 mg, 1.10 mmol, 1.1 equiv) as the coupling partner for 10 h. The crude mixture was concentrated and passed through a plug of silica gel (CH_2_Cl_2_ as eluent) to furnish the desired product as orange yellow oil, which solidified on standing (280 mg, 75% yield, mp 74–75 °C).

From one-pot CHB/Sonogashira coupling: the general procedure B was applied to 3-bromobenzotrifluoride (279 μL, 450 mg, 2.0 mmol, 1 equiv). The borylation step was carried out with HBpin (436 μL, 384 mg, 3.00 mmol, 1.50 equiv) for 3 h. The Sonogashira coupling step was carried out with phenyl acetylene (242 μL, 225 mg, 2.20 mmol, 1.1 equiv) for 5 h. Gradient column chromatography (pentane:dichloromethane 4:1 → pentane:dichloromethane 1:1) furnished the desired product as orange yellow oil, which solidified on standing (473 mg, 64% yield, mp 74–75 °C).

^1^H NMR (CDCl_3_, 300 MHz): δ 8.15 (m, 1 H), 8.00 (m, 1.0 Hz, 1 H), 7.86 (m, 1 H), 7.58–7.49 (m, 2 H), 7.42–7.30 (m, 3 H), 1.37 (s, 12 H, 4 CH_3_ of Bpin). ^13^C-NMR {^1^H} (CDCl_3_, 125 MHz): δ 141.2 (CH), 131.8 (2 CH), 130.8 (q, ^3^*J*_C–F_ = 3.8 Hz, CH), 130.7 (q, ^3^*J*_C–F_ = 3.8 Hz, CH), 130.6 (q, ^2^*J*_C–F_ = 32.5 Hz, C), 128.8 (CH), 128.5 (2 CH), 124.1 (q, ^1^*J*_C–F_ = 273 Hz, CF_3_), 124.0 (C), 122.9 (C), 91.2 (C), 88.0 (C), 84.6 (2 C), 24.9 (4 CH_3_ of Bpin); ^11^B-NMR (CDCl_3_, 96 MHz): δ 30.6; ^19^F-NMR (CDCl_3_, 282 MHz) δ −63.0; FT-IR (neat) ῦ_max_: 2980, 1601, 1493, 1369, 1306, 1277, 1169, 1130, 966, 898, 871, 847, 756, 704, 688 cm^−1^; GC-MS (EI) *m/z* (% relative intensity): M^+^ 372 (100), 357 (10), 286 (18), 272 (12); HRMS (FAB): *m/z* 372.1510 [(M^+^); Calcd for C_21_H_20_BF_3_O_2_: 372.1508].

*3-(Trimethylsilylethynyl)-5-(4,4,5,5-tetramethyl-1,3,2-dioxaborolane-2-yl)-toluene (**13**)*

From Sonogashira coupling of borylated aryl bromide: the general procedure A was applied to the borylated version of 3-bromotoluene (**10**, 297 mg, 1.0 mmol, 1 equiv) with trimethylsilyl acetylene (156 μL, 108 mg, 1.10 mmol, 1.1 equiv) as the coupling partner for 4 h. Column chromatography (pentane/ether 9:1, R*_f_* 0.8) furnished the desired product as yellow oil (194 mg, 62% yield).

From one-pot CHB/Sonogashira coupling: the general procedure B was applied to 3-bromotoluene (122 μL, 171 mg, 1.0 mmol, 1 equiv). The borylation step was carried out with HBpin (218 μL, 192 mg, 1.50 mmol, 1.50 equiv) for 12 h. The Sonogashira coupling step was carried out with trimethylsilyl acetylene (156 μL, 108 mg, 1.10 mmol, 1.1 equiv) for 4 h. Column chromatography (pentane/ether 9:1, R*_f_* 0.8) furnished the desired product as yellow oil (204 mg, 65% yield). 

^1^H NMR (CDCl_3_, 500 MHz): δ 7.74 (m, 1 H), 7.56 (m, 1 H), 7.38 (m, 1 H), 2.31 (m, 3 H), 1.34 (br s, 12 H, 4 CH_3_ of Bpin), 0.22 (s, 9 H, 3 CH_3_ of TMS); ^13^C-NMR {^1^H} (CDCl_3_, 125 MHz): δ 137.3 (C), 135.7 (CH), 135.5 (CH), 135.2 (CH), 122.7 (C), 105.4 (C), 93.8 (C), 84.1 (2 C), 25.0 (4 CH_3_ of Bpin), 21.1 (CH_3_), 0.2 (3 CH_3_ of TMS); ^11^B-NMR (CDCl_3_, 160 MHz): δ 30.2; FT-IR (neat) ῦ_max_: 2978, 2154, 1591, 1383, 1365, 1248, 1145, 966, 848, 760, 706 cm^−1^; GC-MS (EI) *m/z* (% relative intensity): M^+^ 314 (15), 299 (100), 199 (11); HRMS (FAB): *m/z* 314.1875 [(M+); Calcd for C_18_H_27_BO_2_Si: 314.1873].

*3-(Trimethylsilylethynyl)-5-(4,4,5,5-tetramethyl-1,3,2-dioxaborolane-2-yl)-chlorobenzene (**14**)*

From Sonogashira coupling of borylated aryl bromide: the general procedure A was applied to the borylated version of 3-bromochlorobenzene (**11**, 10 g, 31.5 mmol, 1 equiv) with 1,4-diazabicyclo[2.2.2]octane [DABCO] (3.54 g, 31.5 mmol, 1 equiv), allylpalladium chloride dimer (288 mg, 0.788 mmol, 2.5 mol %), P*t-*Bu_3_ (638 mg, 3.15 mmol, 10 mol %), trimethylsilyl acetylene (4.5 mL, 3.09 g, 31.5 mmol, 1 equiv) and acetonitrile (100 mL) for 4 h. After completion, 100 mL of water was added to the reaction mixture. The reaction mixture was extracted with MTBE (50 mL × 3). The combined ether extractions were washed with water (50 mL), followed by brine (50 mL), dried over MgSO_4_ before being concentrated under reduced pressure on a rotary evaporator. Gradient column chromatography (hexanes/ dichloromethane 1:1 → hexanes/dichloromethane 0:1) furnished the desired product as yellow oil. If the oil is left to dry in air, it will dry to a waxy solid that can be scraped and dried under vacuum to a yellow powder (8 g, 76% yield).

From one-pot CHB/Sonogashira coupling: the general procedure B was applied to 3-bromochlorobenzene (118 μL, 191 mg, 1.0 mmol, 1 equiv). The borylation step was carried out with HBpin (218 μL, 192 mg, 1.50 mmol, 1.50 equiv) for 4 h. The Sonogashira coupling step was carried out with trimethylsilyl acetylene (184 μL, 128 mg, 1.30 mmol, 1.3 equiv) for 4 h. Gradient column chromatography (hexanes/ dichloromethane 1:1 → hexanes/dichloromethane 0:1) furnished the desired product as yellow oil (196 mg, 59% yield).

^1^H-NMR (CDCl_3_, 300 MHz): δ 7.78 (dd, *J* = 1.5, 1.0 Hz, 1 H), 7.70 (dd, *J* = 2.2, 1.0 Hz, 1 H), 7.52 (dd, *J* = 2.2, 1.5 Hz, 1 H), 1.34 (br s, 12 H, 4 CH_3_ of Bpin), 0.23 (s, 9 H, 3 CH_3_ of TMS); ^13^C NMR {^1^H} (CDCl_3_, 125 MHz): δ 136.5 (CH), 134.6 (CH), 134.2 (CH), 133.9 (C), 124.6 (C), 103.6 (C), 95.8 (C), 84.5 (2 C), 25.0 (4 CH_3_ of Bpin), 0.0 (3 CH_3_ of TMS); ^11^B-NMR (CDCl_3_, 96 MHz): δ 30.6; FT-IR (neat) ῦ_max_: 2978, 2166, 1562, 1352, 1143, 966, 927, 844, 760, 702 cm^−1^; GC-MS (EI) *m/z* (% relative intensity): M^+^ 334 (8), 320 (100), 219 (10); HRMS (FAB): *m/z* 335.1407 [(M^+^); Calcd for C_17_H_25_BO_2_SiCl: 335.14055].

*3-(Phenylethynyl)-5-(4,4,5,5-tetramethyl-1,3,2-dioxaborolane-2-yl)-toluene (**15**)*

The general procedure B was applied to 3-bromotoluene (122 μL, 171 mg, 1.0 mmol, 1 equiv). The borylation step was carried out with HBpin (290 μL, 256 mg, 2.00 mmol, 2.00 equiv) for 12 h. The Sonogashira coupling step was carried out with phenyl acetylene (121 μL, 112 mg, 1.10 mmol, 1.1 equiv) for 12 h. Column chromatography (pentane/dichloromethane 1:1, R*_f_* 0.8) furnished the desired product as yellow oil, which solidified on standing (193 mg, 61% yield, mp 73–75 °C).

^1^H-NMR (CDCl_3_, 500 MHz): δ 7.83 (m, 1 H), 7.60 (m, 1 H), 7.47–7.50 (m, 2 H), 7.46 (m, 1 H), 7.30–7.34 (m, 3 H), 2.36 (s, 3 H), 1.36 (br s, 12 H, 4 CH_3_ of Bpin); ^13^C NMR {^1^H} (CDCl_3_, 75 MHz): δ 137.4 (C), 135.4 (CH), 135.4 (CH), 134.9 (CH), 131.7 (2 CH), 128.4 (2 CH), 128.2 (CH), 123.7 (C), 123.0 (C), 89.8 (C), 89.3 (C), 84.1 (2 C), 25.0 (4 CH_3_ of Bpin), 21.2 (CH_3_); ^11^B-NMR (C_6_D_6_, 96 MHz): δ 31.7; FT-IR (neat) ῦ_max_: 2976, 1595, 1491, 1417, 1385, 1371, 1317, 1289, 1207, 1143, 966, 852, 756, 706, 690 cm^−1^; GC-MS (EI) *m/z* (% relative intensity): M^+^ 318 (100), 304 (15), 233 (11), 219 (12); HRMS (FAB): *m/z* 318.1794 [(M^+^); Calcd for C_21_H_23_BO_2_: 318.1791].

*3-(Phenylethynyl)-5-(4,4,5,5-tetramethyl-1,3,2-dioxaborolane-2-yl)-anisole (**16**)*

The general procedure B was applied to 3-bromoanisole (254 μL, 374 mg, 2.0 mmol, 1 equiv). The borylation step was carried out with HBpin (580 μL, 512 mg, 4.00 mmol, 2.00 equiv) for 16 h. The Sonogashira coupling step was carried out with phenyl acetylene (286 μL, 266 mg, 2.60 mmol, 1.3 equiv) for 4 h. Gradient column chromatography (hexanes/dichloromethane 1:1 → hexanes/dichloromethane 0:1) furnished the desired product as yellow oil (343 mg, 52% yield).

^1^H-NMR (CDCl_3_, 500 MHz): δ 7.61 (dd, *J* = 1.5, 0.9 Hz, 1 H), 7.54–7.49 (m, 2 H), 7.37–7.31 (m, 3 H), 7.30 (dd, *J* = 2.7, 0.9 Hz, 1 H), 7.15 (dd, *J* = 2.7, 1.5, Hz, 1 H), 3.85 (s, 3 H), 1.35 (br s, 12 H, 4 CH_3_ of Bpin); ^13^C-NMR {^1^H} (CDCl_3_, 125 MHz): δ 159.1 (C), 131.7 (2 CH), 130.8 (CH), 128.5 (2 CH), 128.3 (CH), 124.1 (C), 123.5 (C), 120.0 (CH), 119.8 (CH), 89.38 (C), 89.36 (C), 84.2 (2 C), 55.6 (OCH_3_), 25.0 (4 CH_3_ of Bpin); ^11^B NMR (CDCl_3_, 96 MHz): δ 30.6; FT-IR (neat) ῦ_max_: 2980, 1581, 1373, 1224, 1143, 1057, 966,850, 756, 704 cm^−1^; GC-MS (EI) *m/z* (% relative intensity): M^+^ 334 (100), 319 (10), 276 (6), 248 (15), 234 (21); HRMS (FAB): *m/z* 334.1742 [(M^+^); Calcd for C_21_H_23_BO_3_: 334.1740].

*N,N-Di-methyl-3-(phenylethynyl)-5-(4,4,5,5-tetramethyl-1,3,2-dioxaborolane-2-yl)-aniline (**17**)*

The general procedure B was applied to N,N-dimethyl-3-bromoaniline (400 mg, 2.0 mmol, 1 equiv). The borylation step was carried out with HBpin (580 μL, 512 mg, 4.00 mmol, 2.00 equiv) for 24 h. The Sonogashira coupling step was carried out with phenyl acetylene (242 μL, 225 mg, 2.20 mmol, 1.1 equiv) for 20 h. Column chromatography (pentane/ether 4:1, R*_f_* 0.5) furnished the desired product as yellow oil (488 mg, 70% yield).

^1^H-NMR (C_6_D_6_, 300 MHz): δ 8.03 (dd, *J* = 1.4, 0.8 Hz, 1 H), 7.59–7.50 (m, 2 H), 7.48 (dd, *J* = 2.8, 0.8 Hz, 1 H), 7.12 (dd, *J* = 2.8, 1.4 Hz, 1 H), 7.06-6.96 (m, 3 H), 2.40 (s, 6 H), 1.15 (br s, 12 H, 4 CH_3_ of Bpin); ^13^C-NMR {^1^H} (C_6_D_6_, 75 MHz): δ 150.4 (C), 132.0 (2 CH), 128.6 (2 CH), 128.2 (CH), 127.6 (CH), 124.4 (C), 124.0 (C), 119.6 (CH), 118.4 (CH), 91.5 (C), 89.1 (C), 83.9 (2 C), 40.1 (2 CH_3_), 25.1 (4 CH_3_ of Bpin); ^11^B NMR (CDCl_3_, 96 MHz): δ 31.1; FT-IR (neat) ῦ_max_: 2978, 2930, 2799, 1587, 1489, 1429, 1386, 1269, 1143, 1010, 966, 846, 756, 704, 690 cm^−1^; GC-MS (EI) *m/z* (% relative intensity): M^+^ 347 (100), 289 (2), 247 (10); HRMS (FAB): *m/z* 347.2060 [(M^+^); Calcd for C_22_H_26_BNO_2_: 347.2057].

*3-(Phenylethynyl)-5-(4,4,5,5-tetramethyl-1,3,2-dioxaborolane-2-yl)-chlorobenzene (**18**)*

The general procedure B was applied to 3-bromochlorobenzene (118 μL, 191 mg, 1.0 mmol, 1 equiv). The borylation step was carried out with HBpin (290 μL, 256 mg, 2.00 mmol, 2.00 equiv) for 12 h. The Sonogashira coupling step was carried out with phenyl acetylene (121 μL, 112 mg, 1.10 mmol, 1.1 equiv) for 12 h. Column chromatography (pentane/dichloromethane 4:3, R*_f_* 0.8) furnished the desired product as a light yellow solid (117 mg, 37% yield, mp 45–46 °C).

^1^H-NMR (CDCl_3_, 300 MHz): δ 7.87 (dd, *J* = 1.6, 1.0 Hz, 1 H), 7.74 (dd, *J* = 2.2, 1.0 Hz, 1 H), 7.60 (dd, *J* = 2.2, 1.6 Hz, 1 H), 7.56–7.47 (m, 2 H), 7.40–7.32 (m, 3 H), 1.36 (br s, 12 H, 4 CH_3_ of Bpin); ^13^C NMR {^1^H} (CDCl_3_, 75 MHz): δ 136.2 (CH), 134.4 (CH), 134.1 (C), 133.8 (CH), 131.8 (2 CH), 128.7 (CH), 128.5 (2 CH), 124.9 (C), 123.1 (C), 90.7 (C), 88.2 (C), 84.5 (2 C), 25.0 (4 CH_3_ of Bpin); ^11^B NMR (CDCl_3_, 160 MHz): δ 29.9; FT-IR (neat) ῦ_max_: 2978, 1562, 1412, 1356, 1142, 966, 862, 756, 700, 690 cm^−1^; GC-MS (EI) *m/z* (% relative intensity): M^+^ 338 (100), 340(33), 324 (18), 280 (5), 252 (59); HRMS (FAB): *m/z* 338.1247 [(M^+^); Calcd for C_20_H_20_BClO_2_: 338.1245].

*3-(Phenylethynyl)-5-(4,4,5,5-tetramethyl-1,3,2-dioxaborolane-2-yl)-benzonitrile (**19**)*

The general procedure B was applied to 3-bromobenzonitrile (910 mg, 5.0 mmol, 1 equiv). The borylation step was carried out with HBpin (1.09 mL, 960 mg, 7.5 mmol, 1.5 equiv), [Ir(OMe)(COD)]_2_ (50 mg, 0.075 mmol, 3 mol % Ir), and dtbpy (40 mg, 0.15 mmol, 3 mol %) at room temperature for 12 h. The Sonogashira coupling step was carried out with phenyl acetylene (604 μL, 562 mg, 5.50 mmol, 1.1 equiv) for 24 h. After completion, 20 mL of water were added to the reaction mixture. The reaction mixture was extracted with ether (100 mL). The combined ether extractions were washed with brine (25 mL), followed by water (20 mL) and dried over MgSO_4_. Filtration and concentration under reduced pressure on a rotary evaporator furnished the desired product as a light yellow solid (1.652 g, 71% yield, mp 83–85 °C).

^1^H-NMR (CDCl_3_, 300 MHz): δ 8.16 (dd, *J* = 1.7, 1.1 Hz, 1 H), 8.01 (dd, *J* = 1.7, 1.1 Hz, 1 H), 7.85 (t, *J* = 1.7 Hz, 1 H), 7.59–7.46 (m, 2 H), 7.42–7.31 (m, 3 H), 1.36 (br s, 12 H, 4 CH_3_ of Bpin); ^13^C-NMR {^1^H} (CDCl_3_, 75 MHz): δ 141.8 (CH), 137.5 (CH), 136.9 (CH), 131.9 (2 CH), 129.0 (CH), 128.6 (2 CH), 124.6 (C), 122.6 (C), 118.2 (C), 112.7 (C), 91.9 (C), 87.2 (C), 84.9 (2 C), 25.0 (4 CH_3_ of Bpin); ^11^B NMR (CDCl_3_, 160 MHz): δ 29.7; FT-IR (neat) ῦ_max_: 3061, 2980, 2932, 2231 (s), 2212 (w), 1589, 1491, 1415, 1377, 1329, 1298, 1143, 1122, 966, 897, 848, 756, 698, 690 cm^−1^; GC-MS (EI) *m/z* (% relative intensity): M^+^ 329 (100), 314 (8), 244 (46), 230 (27); HRMS (FAB): *m/z* 330.1668 [(M^+^); Calcd for C_21_H_21_BNO_2_: 330.1665].

*3-(Trimethylsilylethynyl)-5-(4,4,5,5-tetramethyl-1,3,2-dioxaborolane-2-yl)-benzonitrile (**20**)*

The general procedure B was applied to 3-bromobenzonitrile (182 mg, 1.0 mmol, 1 equiv). The borylation step was carried out with B_2_pin_2_ (153 mg, 0.60 mmol, 1.2 equiv of boron), [Ir(OMe)(COD)]_2_ (10 mg, 0.015 mmol, 3 mol % Ir), and dtbpy (8 mg, 0.03 mmol, 3 mol %) at room temperature for 2 h. The Sonogashira coupling step was carried out with trimethylsilyl acetylene (156 μL, 108 mg, 1.10 mmol, 1.1 equiv) for 2 h. Column chromatography (pentane/ethylacetate 9:1, R*_f_* 0.7) furnished the desired product as yellow oil (154 mg, 47% yield).

^1^H-NMR (CDCl_3_, 500 MHz): δ 8.07 (dd, *J* = 1.7, 1.1 Hz, 1 H), 7.98 (dd, *J* = 1.7, 1.1 Hz, 1 H), 7.77 (t, *J* = 1.7 Hz, 1 H), 1.34 (br s, 12 H, 4 CH_3_ of Bpin), 0.24 (s, 9 H, 3 CH_3_ of TMS); ^13^C-NMR {^1^H} (CDCl_3_, 125 MHz): δ 142.1 (CH), 137.7 (CH), 137.3 (CH), 124.3 (C), 118.1 (C), 112.5 (C), 102.5 (C), 97.3 (C), 84.8 (2 C), 25.0 (4 CH_3_ of Bpin), −0.1 (3 CH_3_ of TMS); ^11^B-NMR (CDCl_3_, 96 MHz): δ 30.4; FT-IR (neat) ῦ_max_: 2961, 2235, 2158, 1589, 1369, 1250, 1143, 968, 954, 846, 760, 700 cm^−1^; GC-MS (EI) *m/z* (% relative intensity): M^+^ 325 (3), 311 (100), 210 (3); HRMS (FAB): *m/z* 326.1748 [(M^+^); Calcd for C_18_H_25_BO_2_SiN: 326.17477].

*3-(Phenylethynyl)-5-(4,4,5,5-tetramethyl-1,3,2-dioxaborolane-2-yl)-o-xylene (**21**)*

The general procedure B was applied to 3-bromo-o-xylene (136 μL, 185 mg, 1.0 mmol, 1 equiv). The borylation step was carried out with HBpin (290 μL, 256 mg, 2.00 mmol, 2.00 equiv) for 10 h. The Sonogashira coupling step was carried out with phenyl acetylene (143 μL, 132 mg, 1.30 mmol, 1.3 equiv) for 18 h. Column chromatography (pentane/dichloromethane 1:2, R*_f_* 0.8) furnished the desired product as a yellow solid (255 mg, 77% yield, mp 104–105 °C).

^1^H NMR (CDCl_3_, 500 MHz): δ 7.90 (m, 1 H), 7.59 (m, 1 H), 7.57–7.53 (m, 2 H), 7.40–7.30 (m, 3 H), 2.52 (s, 3 H), 2.33 (s, 3 H), 1.38 (br s, 12 H, 4 CH_3_ of Bpin); ^13^C NMR {^1^H} (CDCl_3_, 125 MHz): δ 141.8 (C), 136.6 (CH), 136.2 (C), 136.0 (CH), 131.5 (2 CH), 128.4 (2 CH), 128.1 (CH), 123.9 (C), 123.0 (C), 92.9 (C), 89.1 (C), 83.9 (2 C), 25.0 (4 CH_3_ of Bpin), 20.2 (CH_3_), 17.9 (CH_3_); ^11^B NMR (CDCl_3_, 96 MHz): δ 30.7; FT-IR (neat) ῦ_max_: 2978, 1398, 1389, 1143, 966, 854, 756, 686 cm^−1^; GC-MS (EI) *m/z* (% relative intensity): M^+^ 332 (100), 318 (14), 275 (6), 247 (8), 232 (20), 218 (12); HRMS (FAB): *m/z* 332.1948 [(M^+^); Calcd for C_22_H_25_BO_2_: 332.19477].

*2-(Phenylethynyl)-5-(4,4,5,5-tetramethyl-1,3,2-dioxaborolane-2-yl)-m-xylene (**22**)*

The general procedure B was applied to 2-bromo-m-xylene (134 μL, 185 mg, 1.0 mmol, 1 equiv). The borylation step was carried out with HBpin (290 μL, 256 mg, 2.00 mmol, 2.00 equiv) for 4 h. The Sonogashira coupling step was carried out with phenyl acetylene (143 μL, 132 mg, 1.30 mmol, 1.3 equiv) for 40 h. Gradient column chromatography (hexanes/dichloromethane 2:1 → hexanes: dichloromethane 0:1) furnished the desired product as yellow oil (233 mg, 70% yield).

^1^H-NMR (CDCl_3_, 500 MHz): δ 7.57–7.53 (m, 2 H), 7.53 (m, 2 H), 7.39–7.32 (m, 3 H), 2.52 (t, *J* = 0.7 Hz, 6 H), 1.36 (br s, 12 H, 4 CH_3_ of Bpin); ^13^C-NMR {^1^H} (CDCl_3_, 125 MHz): δ 139.5 (2 C), 133.0 (2 CH), 131.6 (2 CH), 128.5 (CH), 128.4 (CH), 126.0 (C), 123.8 (C), 99.2 (C), 87.5 (C), 84.0 (2 C), 25.0 (4 CH_3_ of Bpin), 21.0 (2 CH_3_); ^11^B-NMR ((CD_3_)_2_CO, 96 MHz): δ 30.6; FT-IR (neat) ῦ_max_: 2978, 1606, 1385, 1365, 1315, 1238, 1143, 856, 756, 686 cm^−1^; GC-MS (EI) *m/z* (% relative intensity): M^+^ 332 (100), 318 (5), 247 (22), 233 (16), 218 (9); HRMS (FAB): *m/z* 332.1950 [(M^+^); Calcd for C_22_H_25_BO_2_: 332.1948].

*1,3-Bis-(trimethylsilylethynyl)-5-(4,4,5,5-tetramethyl-1,3,2-dioxaborolane-2-yl)-benzene (**23**)*

The general procedure B was applied to 1,3-di-bromobenzene (121 μL, 236 mg, 1.0 mmol, 1 equiv). The borylation step was carried out with HBpin (218 μL, 218 mg, 1.50 mmol, 1.50 equiv) for 8 h. The Sonogashira coupling step was carried out with trimethylsilyl acetylene (312 μL, 216 mg, 2.20 mmol, 2.2 equiv) for 2 h. Column chromatography (pentane/dichloromethane 2:1, R*_f_* 0.8) furnished the desired product as yellow oil (212 mg, 54% yield).

^1^H-NMR (CDCl_3_, 500 MHz): δ 7.84 (d, *J* = 1.7 Hz, 2 H), 7.64 (t, *J* = 1.7 Hz, 1 H), 1.32 (br s, 12 H, 4 CH_3_ of Bpin), 0.22 (s, 18 H, 6 CH_3_ of 2 TMS); ^13^C-NMR {^1^H} (CDCl_3_, 125 MHz): δ 138.1 (2 CH), 137.6 (CH), 123.1 (2 C), 104.2 (C), 94.9 (C), 84.3 (2 C), 25.0 (4 CH_3_ of Bpin), 0.1 (6 CH_3_ of 2 TMS); ^11^B NMR (CDCl_3_, 160 MHz): δ 29.7 (trace unidentified organoboronate at δ 33.9); FT-IR (neat) ῦ_max_: 2961, 2899, 2154, 1583, 1412, 1371, 1250, 976, 844, 760, 702 cm^−1^; GC-MS (EI) *m/z* (% relative intensity): M^+^ 396 (14), 382 (100), 282 (7); HRMS (FAB): *m/z* 396.2116 [(M^+^); Calcd for C_22_H_33_BO_2_Si: 396.2112].

*1,2-Bis-(trimethylsilylethynyl)-4-(4,4,5,5-tetramethyl-1,3,2-dioxaborolane-2-yl)-benzene (**24**).*

The general procedure B was applied to 1,2-di-bromobenzene (121 μL, 236 mg, 1.0 mmol, 1 equiv). The borylation step was carried out with HBpin (218 μL, 192 mg, 1.50 mmol, 1.50 equiv) for 16 h. The Sonogashira coupling step was carried out with [DABCO] (449 mg, 4.0 mmol, 4 equiv) and trimethylsilyl acetylene (340 μL, 236 mg, 2.40 mmol, 2.4 equiv) for 12 h. Gradient column chromatography (hexanes/dichloromethane 1:1 → hexanes: dichloromethane 0:1) furnished the desired product as a light yellow solid (226 mg, 57% yield, mp 123–124 °C).

^1^H-NMR (CDCl_3_, 500 MHz): δ 7.91 (dd, *J* = 1.3, 0.6 Hz, 1 H), 7.64 (dd, *J* = 7.7, 1.3 Hz, 1 H), 7.45 (dd, *J* = 7.7, 0.6 Hz, 1 H), 1.33 (br s, 12 H, 4 CH_3_ of Bpin), 0.27 (s, 9 H, 3 CH_3_ of 2 TMS), 0.26 (s, 9 H, 3 CH_3_ of 2 TMS); ^13^C-NMR {^1^H} (CDCl_3_, 125 MHz): δ 138.9 (CH), 134.0 (CH), 131.6 (CH), 128.2 (C), 125.3 (C), 103.5 (C), 103.4 (C), 100.0 (C), 98.4 (C), 84.3 (2 C), 25.0 (4 CH_3_ of Bpin), 0.20 (3 CH_3_ of 2 TMS), 0.16 (3 CH_3_ of 2 TMS); ^11^B-NMR ((CD_3_)_2_CO, 96 MHz): δ 31.0; FT-IR (neat) ῦ_max_: 2978, 2961, 2899, 2157, 1599, 1390, 1356, 1250, 964, 924, 844, 760, 684 cm^−1^; GC-MS (EI) *m/z* (% relative intensity): M^+^ 396 (88), 381 (57), 339 (18), 282 (100); HRMS (FAB): *m/z* 396.2119 [(M^+^); Calcd for C_22_H_33_BO_2_Si: 396.2112].

*1,2-Bis-(phenylethynyl)-4-(4,4,5,5-tetramethyl-1,3,2-dioxaborolane-2-yl)-benzene (**25**)*

The general procedure B was applied to 1,2-di-bromobenzene (121 μL, 236 mg, 1 mmol, 1 equiv). The borylation step was carried out with B_2_pin_2_ (153 mg, 0.60 mmol, 1.2 equiv of boron), [Ir(OMe)(COD)]_2_ (10 mg, 0.015 mmol, 3 mol % Ir), dtbpy (8 mg, 0.03 mmol, 3 mol %) in THF (2 mL) at 80 ºC for 8 h. The Sonogashira coupling step was carried out with [DABCO] (449 mg, 4.0 mmol, 4 equiv) and phenyl acetylene (242 μL, 225 mg, 2.20 mmol, 2.2 equiv) for 13 h. Column chromatography (chloroform, R*_f_* 0.9) furnished the desired product as yellow oil (296 mg, 73% yield).

^1^H-NMR (CDCl_3_, 500 MHz) δ 8.08 (d, *J* = 1.0 Hz, 1H), 7.77 (dd, *J* = 7.8, 1.2 Hz, 1H), 7.66 – 7.57 (m, 5H), 7.37 (dt, *J* = 5.4, 2.4 Hz, 6H), 1.39 (s, 12H); ^13^C-NMR {^1^H} (CDCl_3_, 125 MHz): δ 138.4 (CH), 134.0 (CH), 131.8 (2 CH), 131.7 (2 CH), 131.1 (CH), 128.7 (CH), 128.50 (2 CH), 128.48 (2 CH), 128.46 (CH), 128.21 (C), 125.3 (C), 123.5 (C), 123.3 (C), 95.0 (C), 93.6 (C), 88.7 (C), 88.5 (C), 84.3 (2 C), 25.0 (4 CH_3_ of Bpin); ^11^B-NMR (CDCl_3_, 160 MHz): δ 30.1; FT-IR (neat) ῦ_max_: 3059, 2978, 2930, 2214, 1599, 1491, 1400, 1358, 1143, 1107, 964, 916, 854, 756, 688 cm^−1^; MS (EI) *m/z* (% relative intensity): M^+^ 404 (88), 389 (3), 318 (34), 304 (85), 276 (50); HRMS (FAB): *m/z* 404.1950 [(M^+^); Calcd for C_28_H_25_BO_2_: 404.1948].

## 4. Conclusions

In conclusion, alkyne groups are not always compatible with traditional CHB conditions and direct synthesis of borylated aryl alkynes is challenging. However, taking advantage of the tolerance of aryl bromides toward CHB, we have developed an efficient one-pot aromatic C–H activation borylation/Sonogashira coupling protocol for the synthesis of borylated aromatic alkynes. This methodology tolerates a variety of functional groups and several borylated alkynes were prepared in good to high yields. Boronic esters as well as alkynes have a variety of applications in medicinal chemistry, polymers, material science, etc. Boronic esters can serve as sensors for carbohydrates, protecting groups for polymers and sugars, bioactive functional groups or versatile precursors for more complex molecules to name some applications [2]. Introduction of an alkyne functionality to the aromatic ring can extent conjugation and change electronic properties (e.g., fluorescence [40]) or geometrical features (e.g., crystal arrangements [38]) of the molecules. Taking advantage of both functionalities (alkyne and boronic ester) in the same ring can result in useful intermediates, we anticipate that our report will facilitate the synthesis of these compounds and the examination of their properties. 

## Figures and Tables

**Table 1:** Scope of one-pot CHB/Sonagashira cross-coupling reaction^a.^

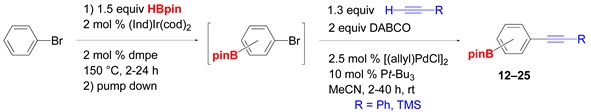					
Entry	Reagent	Product	Entry	Reagent	Product
1	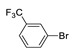	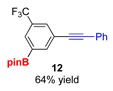	8^b^	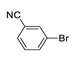	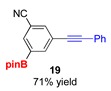
2	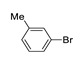	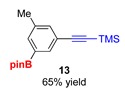	9^b,c^	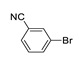	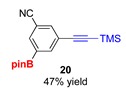
3	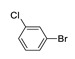	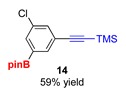	10	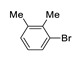	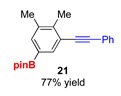
4	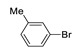	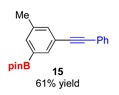	11	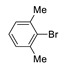	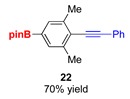
5	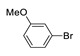	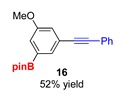	12	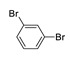	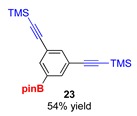
6	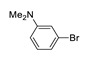	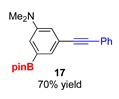	13	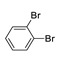	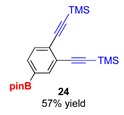
7	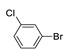	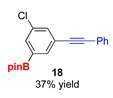	14	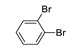	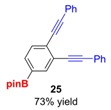

^a^ See Materials and Methods (Section 3) for experimental details and Supplementary Materials for spectral data. ^b^ 3 mol % [Ir(cod)OMe]_2_/dtbpy was used for borylation. ^c^ Borylation was carried out with 0.6 equiv of B_2_pin_2._

## Supplementary Materials

The following are available online: Spectral data for the borylated products.
