# Supplementary material for: One-Pot Iridium Catalyzed C–H Borylation/Sonogashira Cross-Coupling: Access to Borylated Aryl Alkynes
Source: Molecules. 2020 Apr 10;25(7):1754. doi: 10.3390/molecules25071754 (PMC7181282; doi:10.3390/molecules25071754)

One-pot Iridium Catalyzed C–H Borylation/ Sonogashira Cross-Coupling: Access to Borylated Aryl Alkynes

Supporting Information

Ghayoor A. Chotana,[1] Jose R. Montero Bastidas,[1] Susanne L. Miller,[2] Milton R. Smith, III,[1*] and Robert E. Maleczka, Jr.[1*]

[1]Department of Chemistry, Michigan State University, East Lansing, MI 48824-1322, USA
[2]BoroPharm Inc., 39555 Orchard Hill Place, Suite 600, Novi, MI 48375, USA
*Correspondence: maleczka@chemistry.msu.edu (R.E.M.); smithmil@msu.edu (M.R.S.)

Table of Contents
NMR spectrums of compounds reported in Table 1

Entry 1: ^{1}H NMR of 12 ($CDCl_3$, 300 MHz) S4
Entry 1: ^{13}C NMR of 12 ($CDCl_3$, 126 MHz) S5
Entry 1: ^{11}B NMR of 12 (C_6D_6, 96 MHz) S6
Entry 2: ^{1}H NMR of 13 ($CDCl_3$, 500 MHz) S7
Entry 2: ^{13}C NMR of 13 ($CDCl_3$, 126 MHz) S8
Entry 2: ^{11}B NMR of 13 ($CDCl_3$, 160 MHz) S9
Entry 3: ^{1}H NMR of 14 ($CDCl_3$, 300 MHz) S10
Entry 3: ^{13}C NMR of 14 ($CDCl_3$, 126 MHz) S11
Entry 3: ^{11}B NMR of 14 ($CDCl_3$, 160 MHz) S12

Entry 4: ^{1}H NMR of 15 ($CDCl_3$, 300 MHz) S13
Entry 4: ^{13}C NMR of 15 ($CDCl_3$, 75 MHz) S14
Entry 4: ^{11}B NMR of 15 (C_6D_6, 96 MHz).......... S15
Entry 5: ^{1}H NMR of 16 ($CDCl_3$, 500 MHz) S16
Entry 5: ^{13}C NMR of 16 ($CDCl_3$, 126 MHz) S17
Entry 5: ^{11}B NMR of 16 ($(CD_3)_2CO$, 96 MHz) S18
Entry 6: ^{1}H NMR of 17 (C_6D_6, 300 MHz).......... S19
Entry 6: ^{13}C NMR of 17 (C_6D_6, 75 MHz).......... S20
Entry 6: ^{11}B NMR of 17 (C_6D_6, 96 MHz).......... S21
Entry 7: ^{1}H NMR of 18 ($CDCl_3$, 300 MHz) S22
Entry 7: ^{13}C NMR of 18 ($CDCl_3$, 75 MHz) S23
Entry 7: ^{11}B NMR of 18 ($CDCl_3$, 160 MHz) S24
Entry 8: ^{1}H NMR of 19 ($CDCl_3$, 300 MHz) S25
Entry 8: ^{13}C NMR of 19 ($CDCl_3$, 75 MHz) S26
Entry 8: ^{11}B NMR of 19 ($CDCl_3$, 160 MHz) S27
Entry 9: ^{1}H NMR of 20 ($CDCl_3$, 500 MHz) S28
Entry 9: ^{13}C NMR of 20 ($CDCl_3$, 126 MHz) S29
Entry 9: ^{11}B NMR of 20 ($CDCl_3$, 160 MHz) S30
Entry 10: ^{1}H NMR of 21 ($CDCl_3$, 500 MHz) S31
Entry 10: ^{13}C NMR of 21 ($CDCl_3$, 126 MHz) S32
Entry 10: ^{11}B NMR of 21 ($CDCl_3$, 160 MHz) S33

Entry 11: ^{1}H NMR of 22 ($CDCl_3$, 500 MHz) S34
Entry 11: ^{13}C NMR of 22 ($CDCl_3$, 126 MHz) S35
Entry 11: ^{11}B NMR of 22 ($(CD_3)_2CO$, 96 MHz) S36
Entry 12: ^{1}H NMR of 23 ($CDCl_3$, 500 MHz) S37
Entry 12: ^{13}C NMR of 23 ($CDCl_3$, 126 MHz) S38
Entry 12: ^{11}B NMR of 23 ($CDCl_3$, 160 MHz) S39
Entry 13: ^{1}H NMR of 24 ($CDCl_3$, 500 MHz) S40
Entry 13: ^{13}C NMR of 24 ($CDCl_3$, 126 MHz) S41
Entry 13: ^{11}B NMR of 24 ($(CD_3)_2CO$, 96 MHz) S42
Entry 14: ^{1}H NMR of 25 ($CDCl_3$, 500 MHz) S43
Entry 14: ^{13}C NMR of 25 ($CDCl_3$, 126 MHz) S44
Entry 14: ^{11}B NMR of 25 ($CDCl_3$, 160 MHz) S45

Entry 1: ^{1}H NMR of 12 ($CDCl_3$, 300 MHz)

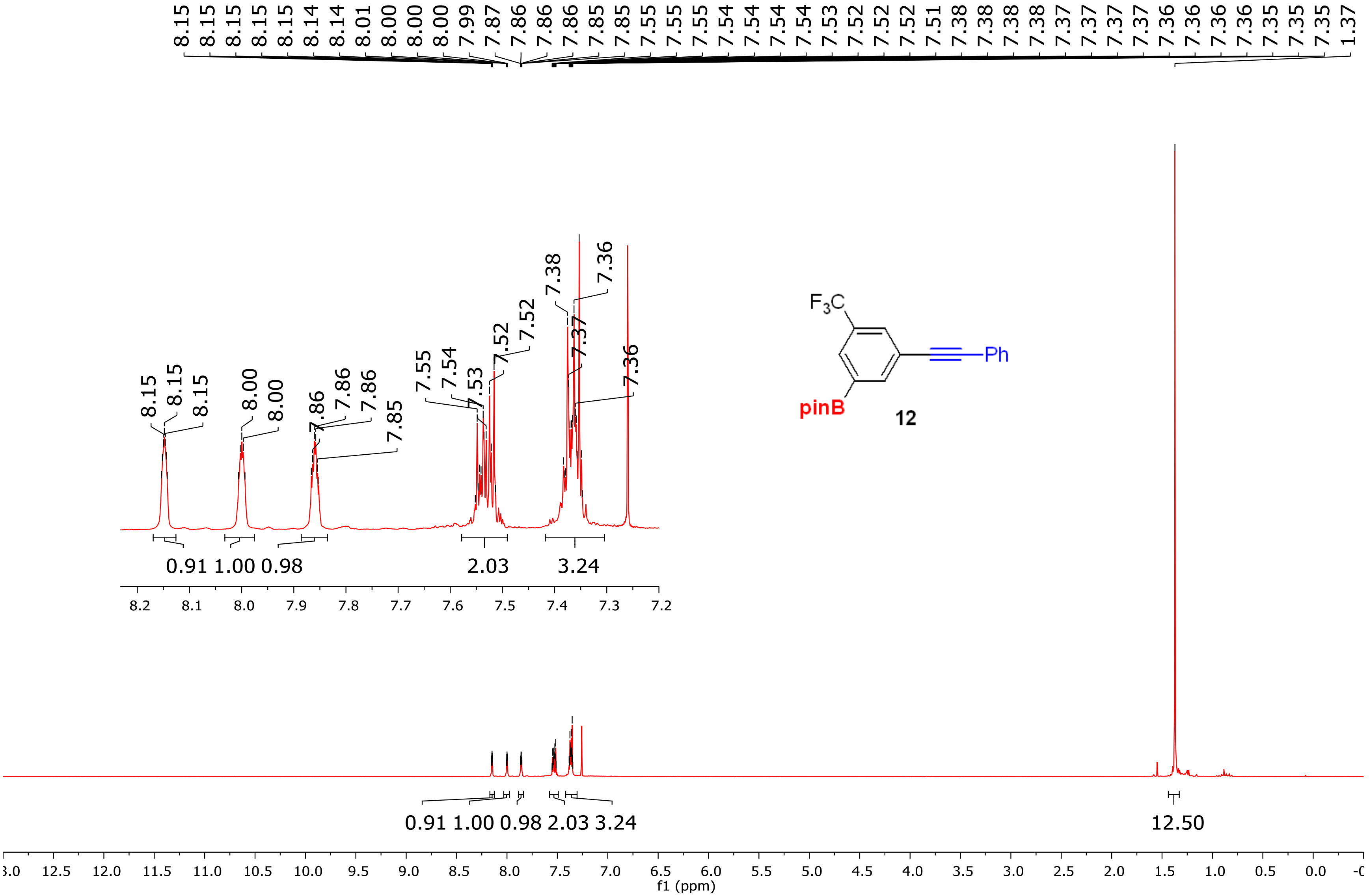

Entry 1: ^{13}C NMR of 12 ($CDCl_3$, 126 MHz)

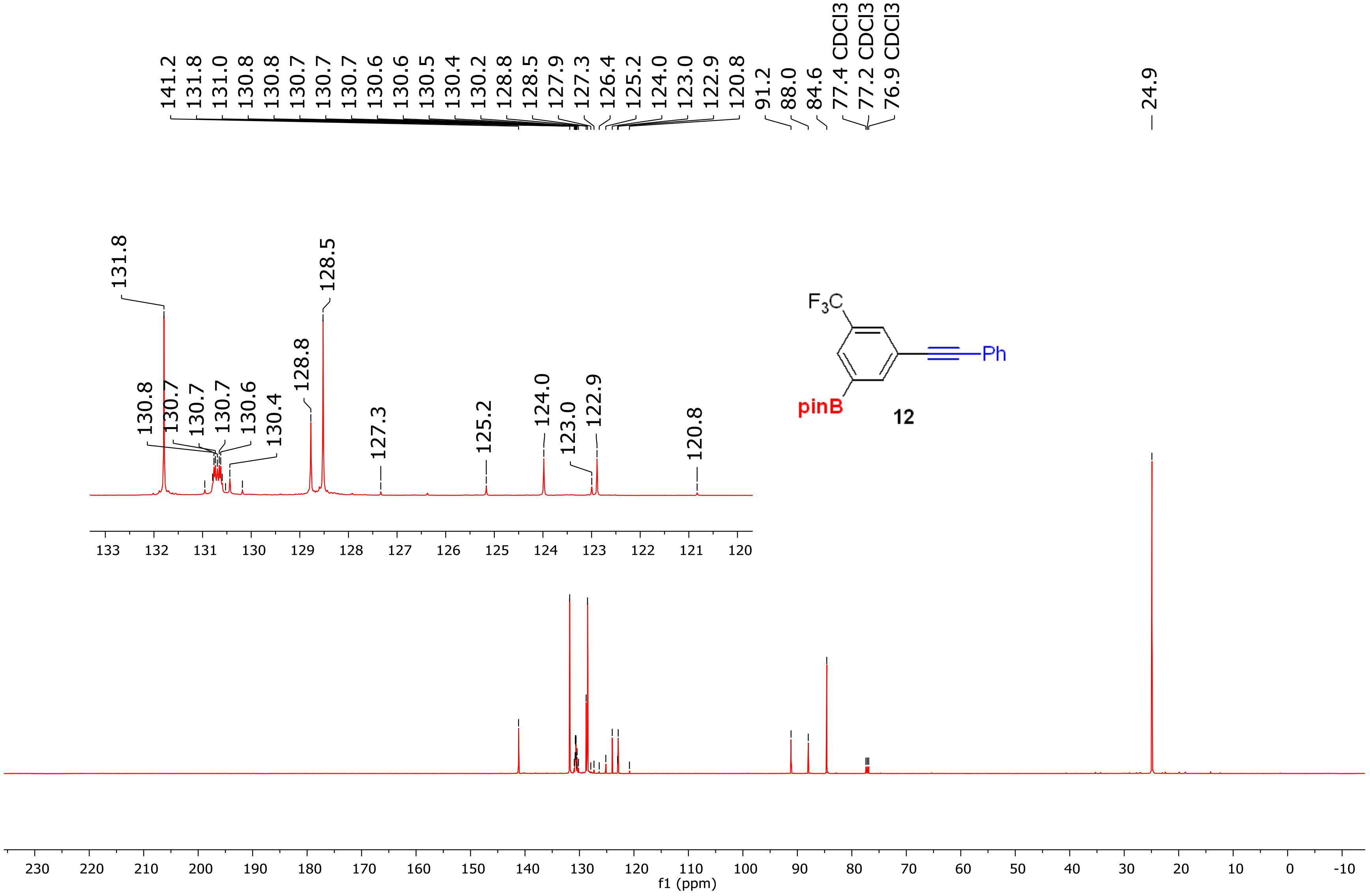

Entry 1: ^{11}B NMR of 12 (C_6D_6, 96 MHz)

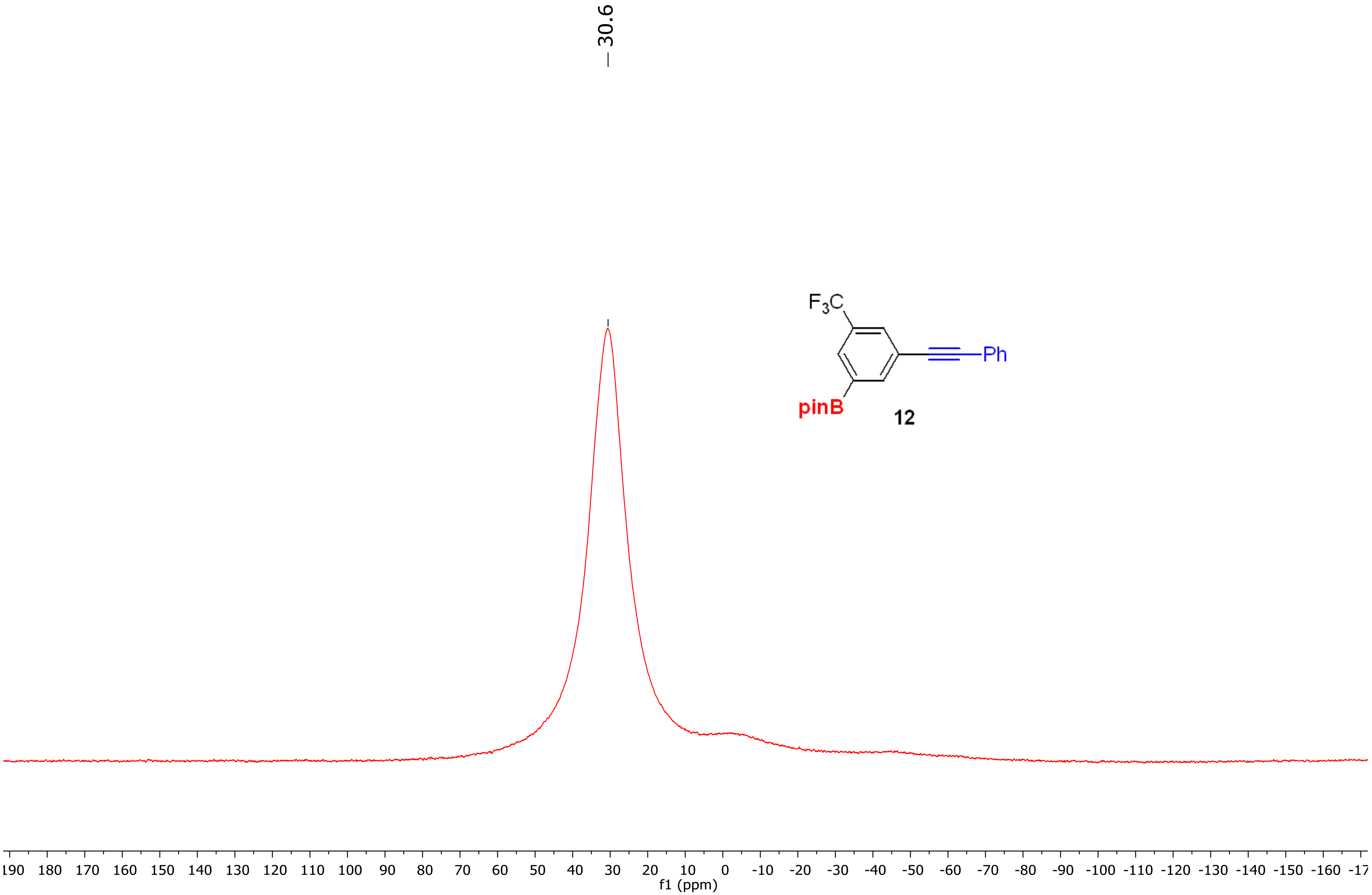

Entry 2: ^{1}H NMR of 13 ($CDCl_3$, 500 MHz)

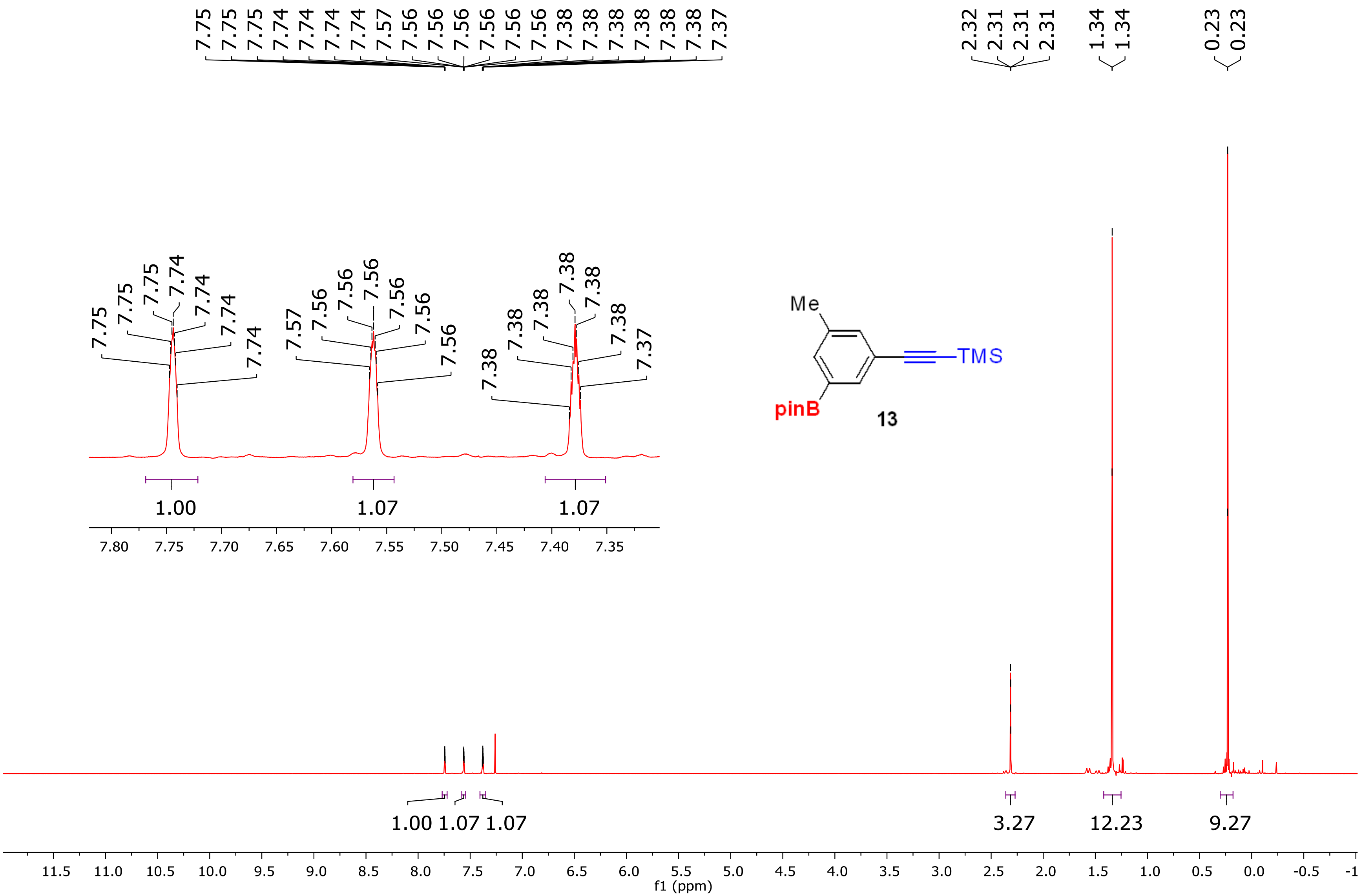

Entry 2: ^{13}C NMR of 13 ($CDCl_3$, 126 MHz)

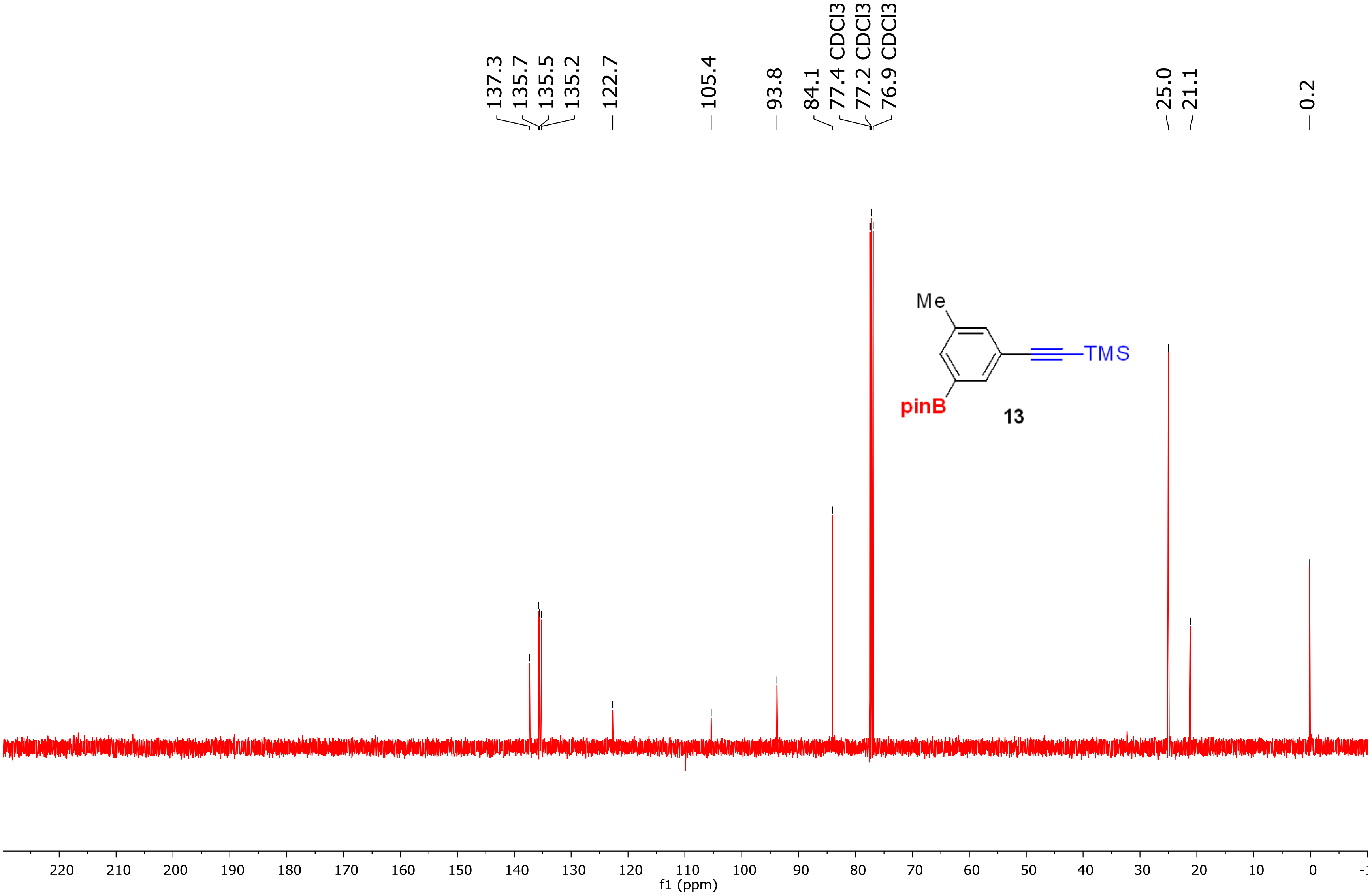

Entry 2: ^{11}B NMR of 13 ($CDCl_3$, 160 MHz)

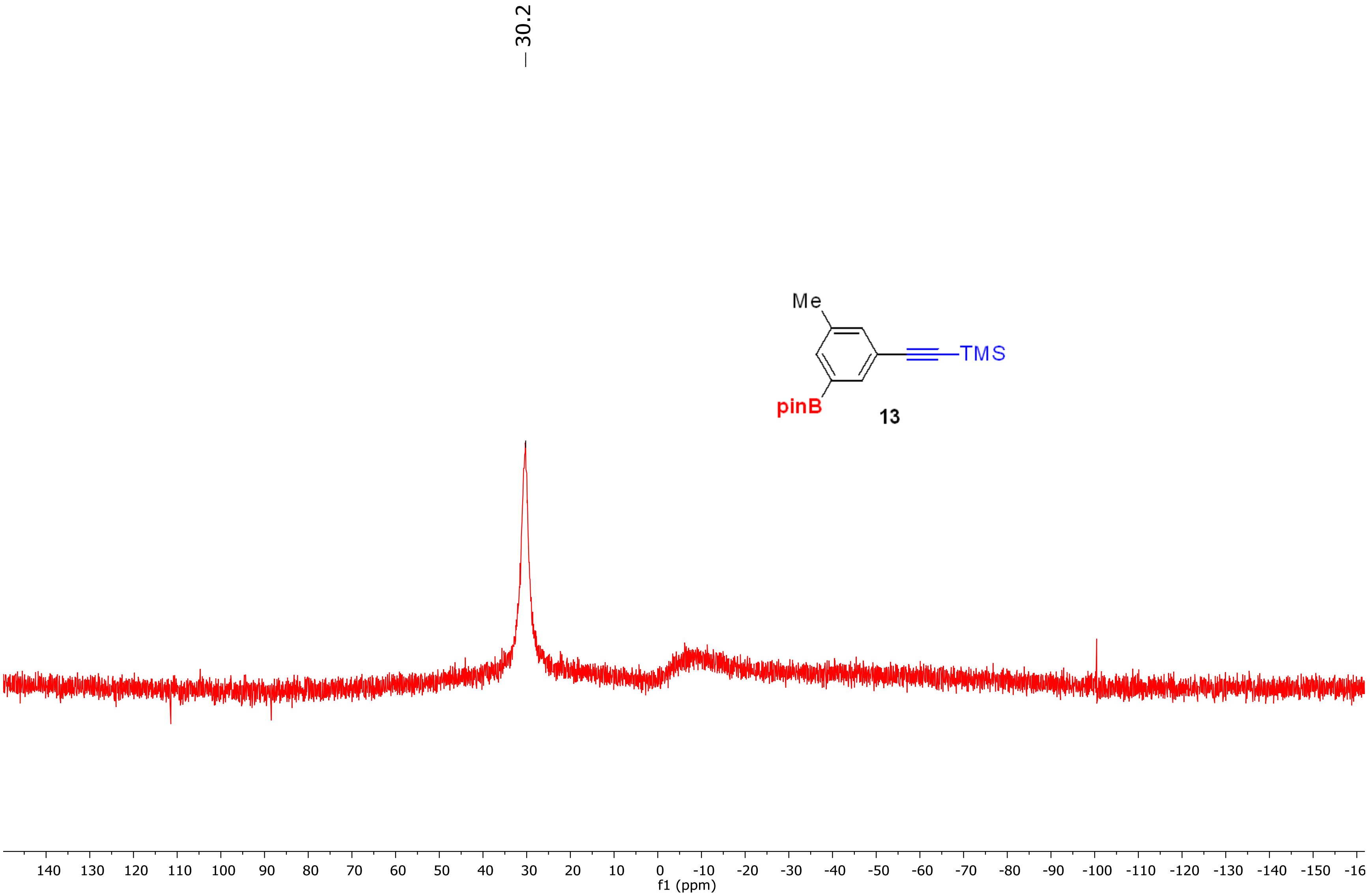

Entry 3: ^{1}H NMR of 14 ($CDCl_3$, 300 MHz)

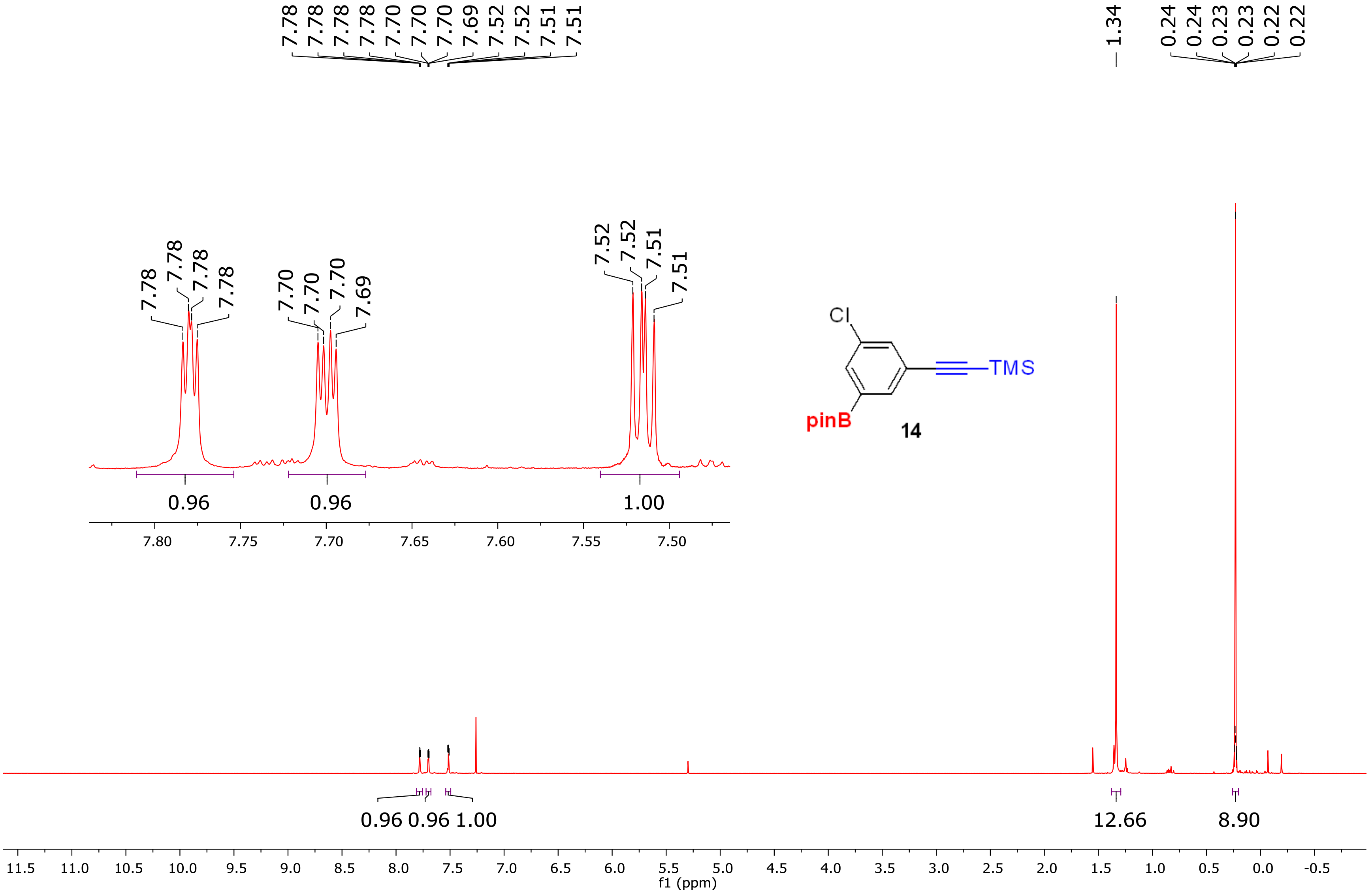

Entry 3: ^{13}C NMR of 14 ($CDCl_3$, 126 MHz)

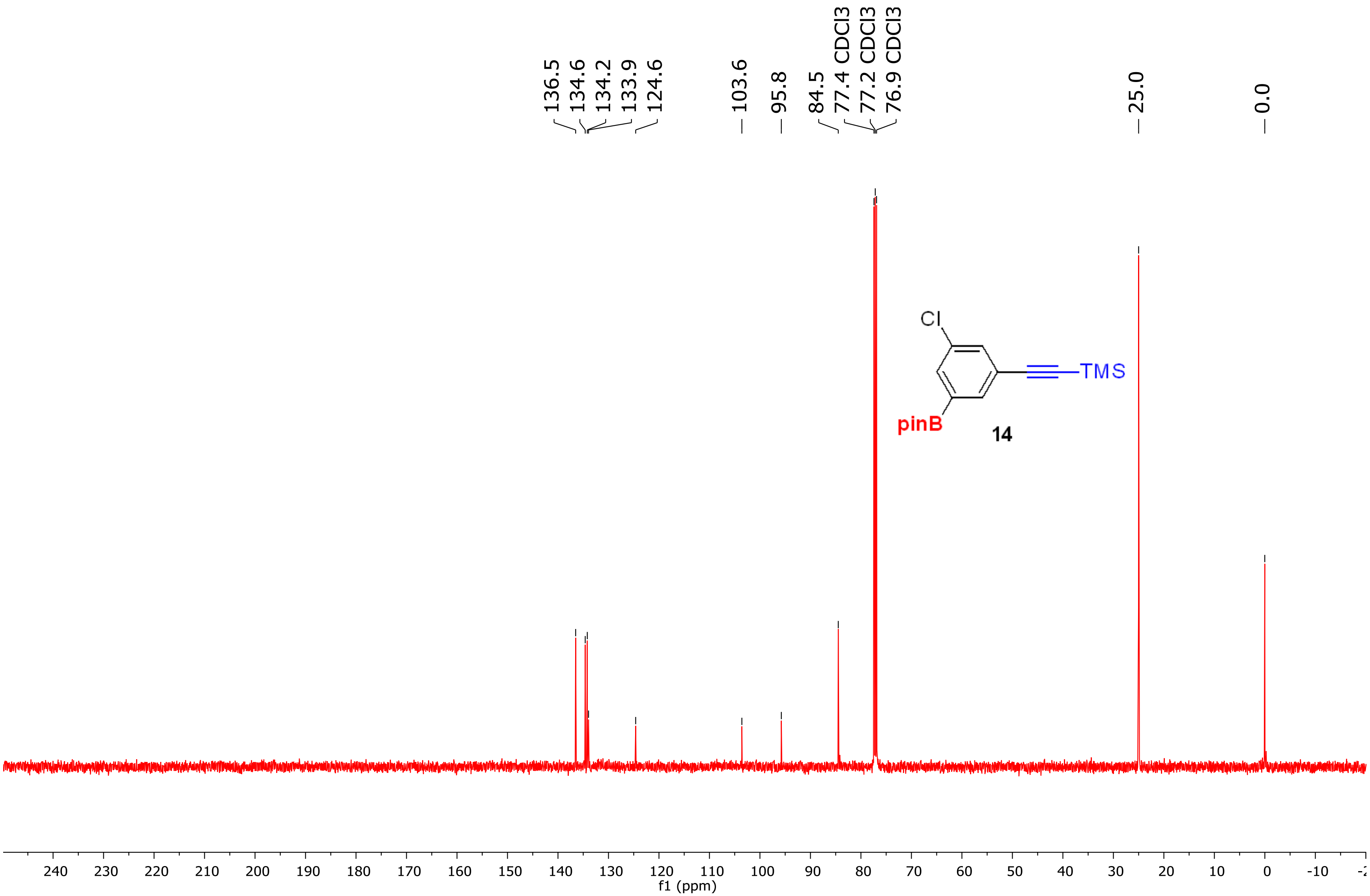

Entry 3: ^{11}B NMR of 14 ($CDCl_3$, 160 MHz)

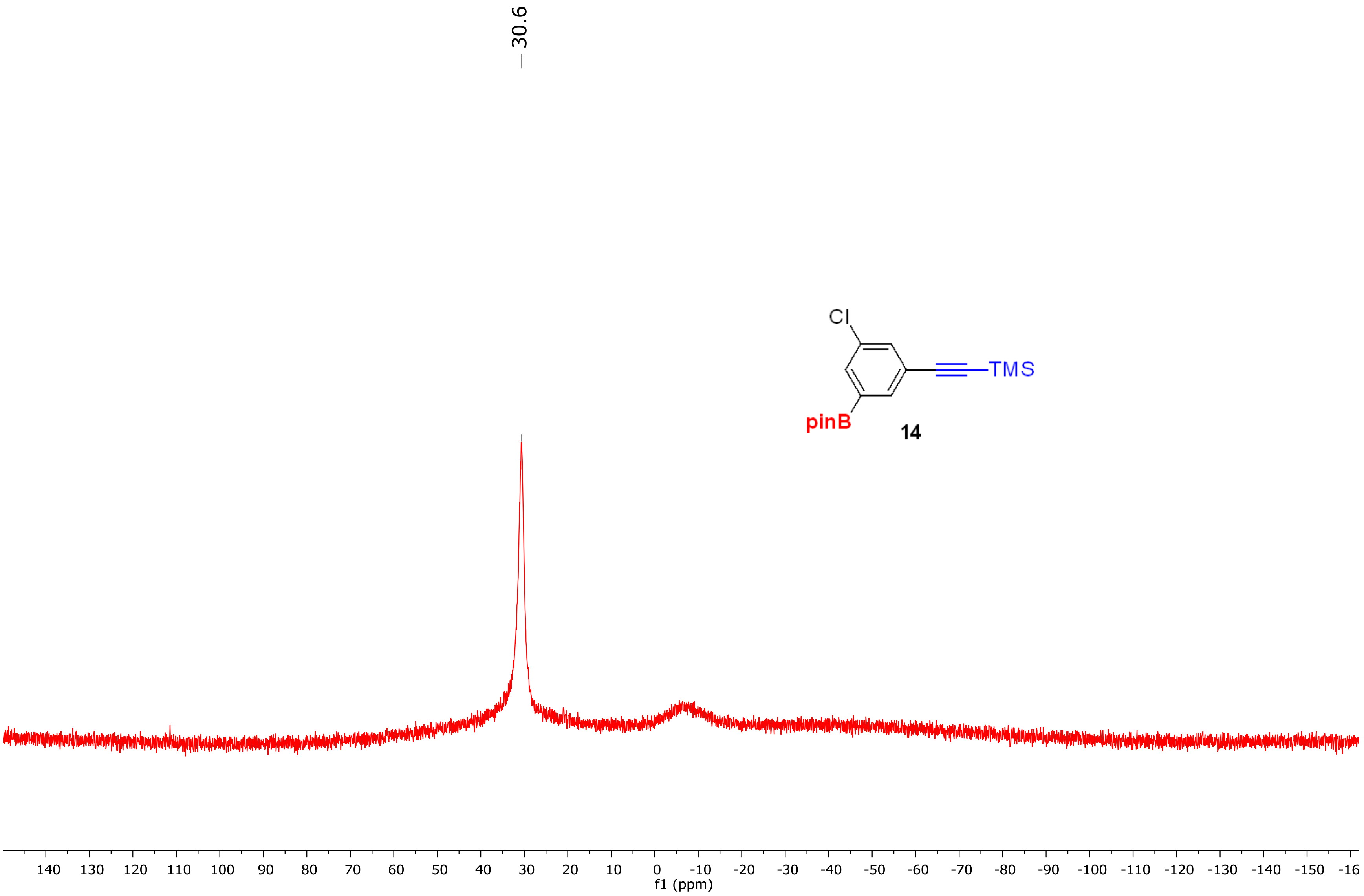

Entry 4: ^{1}H NMR of 15 ($CDCl_3$, 300 MHz)

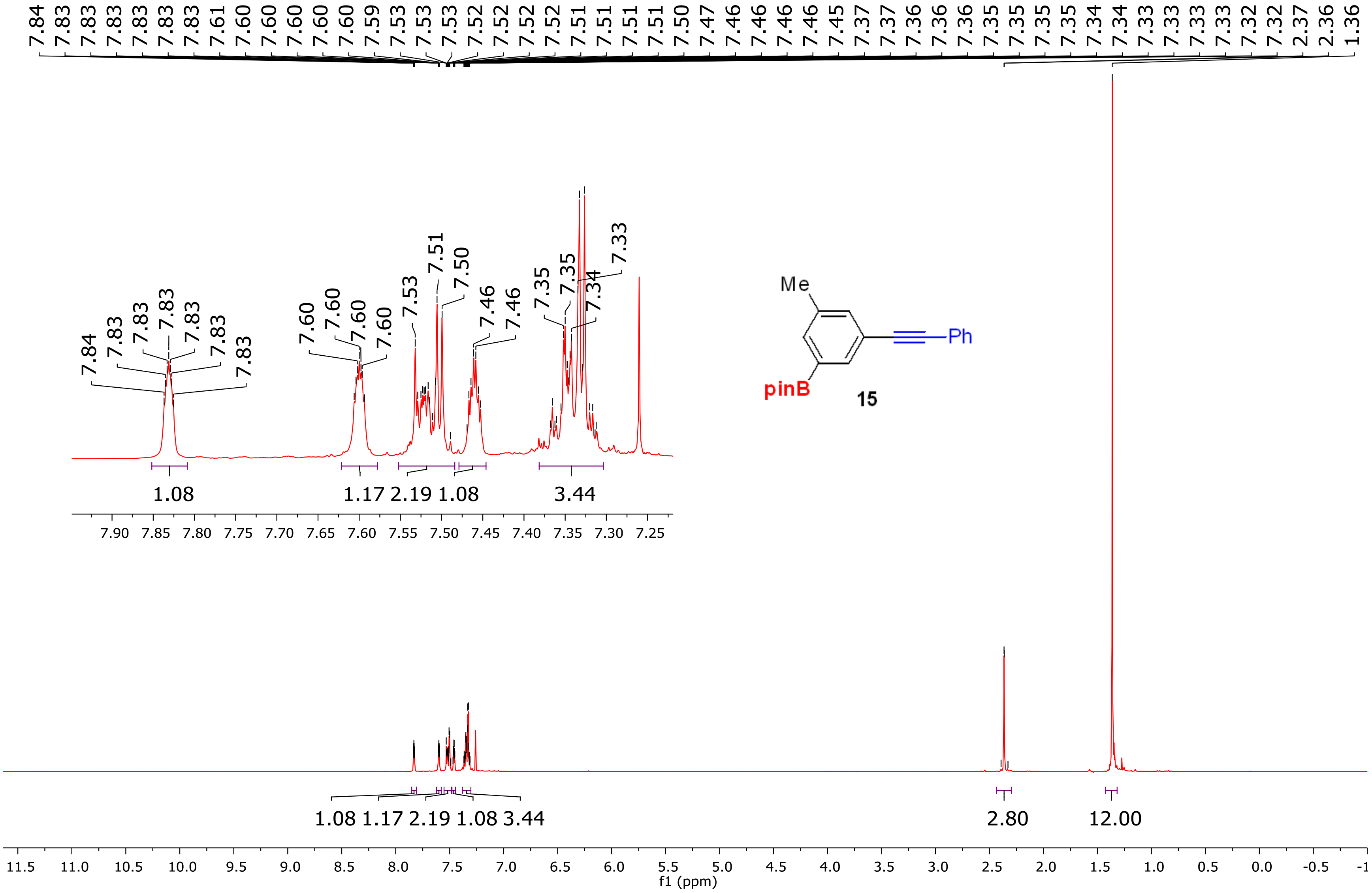

Entry 4: ^{13}C NMR of 15 ($CDCl_3$, 75 MHz)

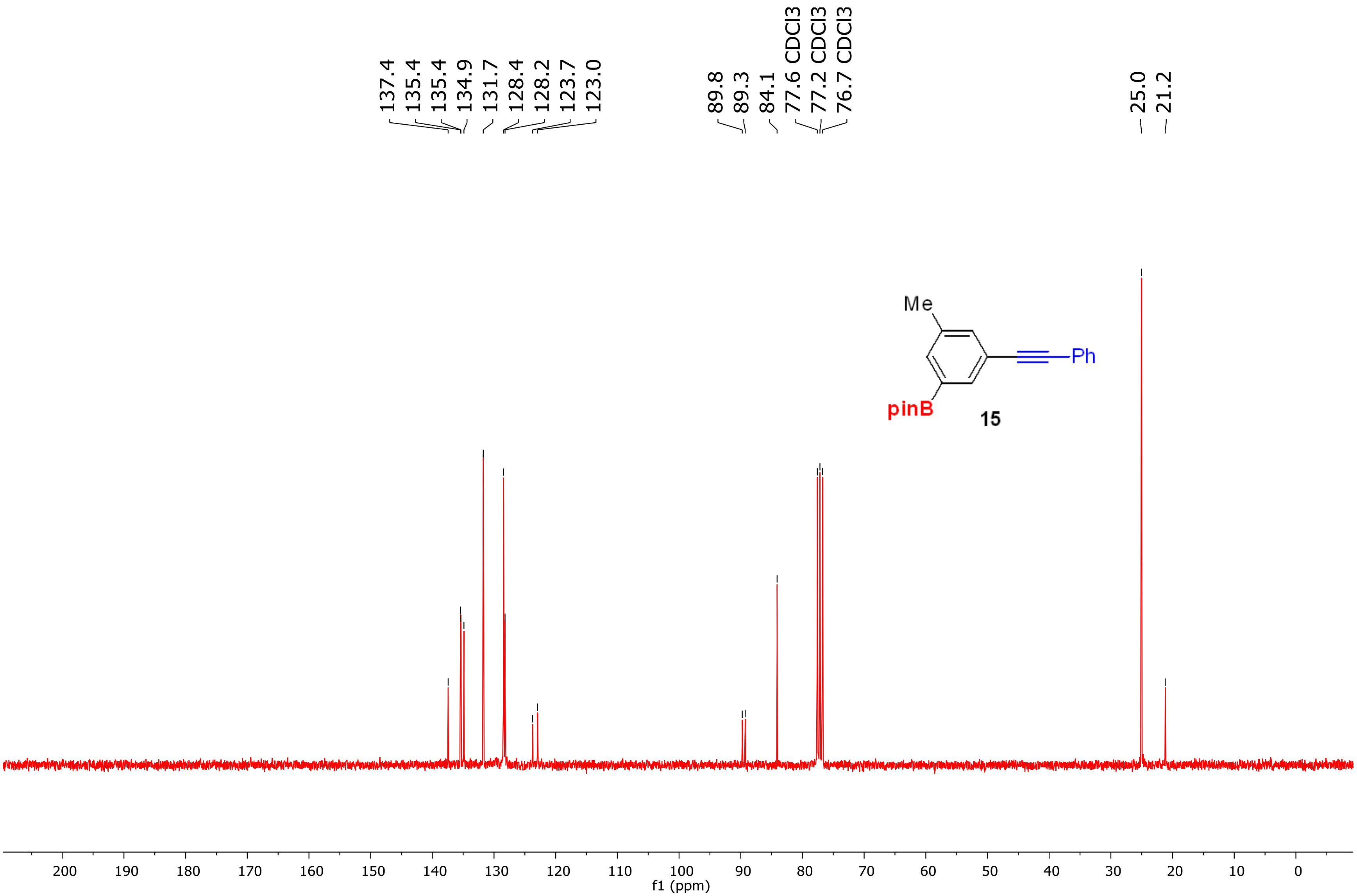

Entry 4: ^{11}B NMR of 15 (C_6D_6, 96 MHz)

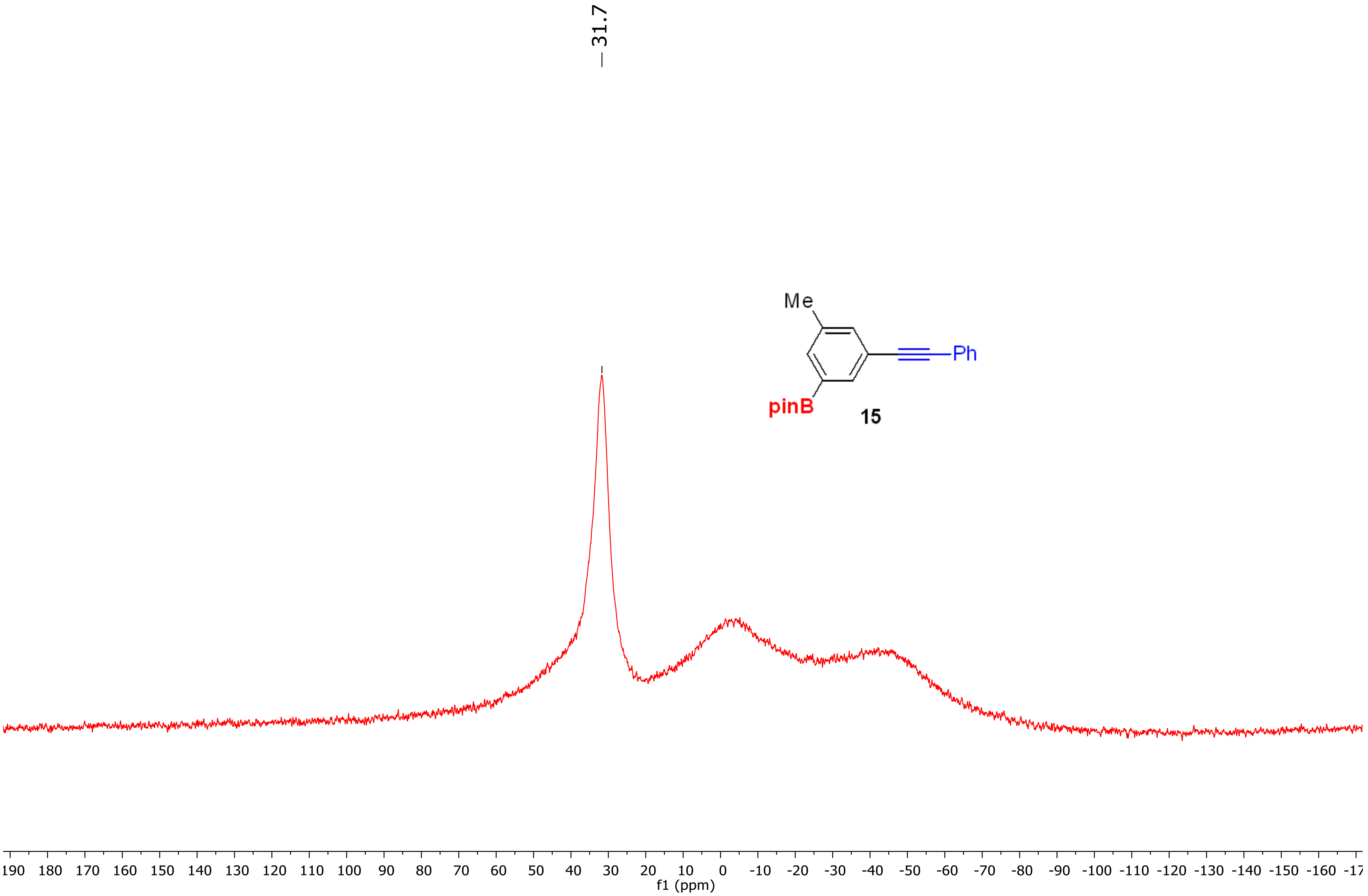

Entry 5: ^{1}H NMR of 16 ($CDCl_3$, 500 MHz)

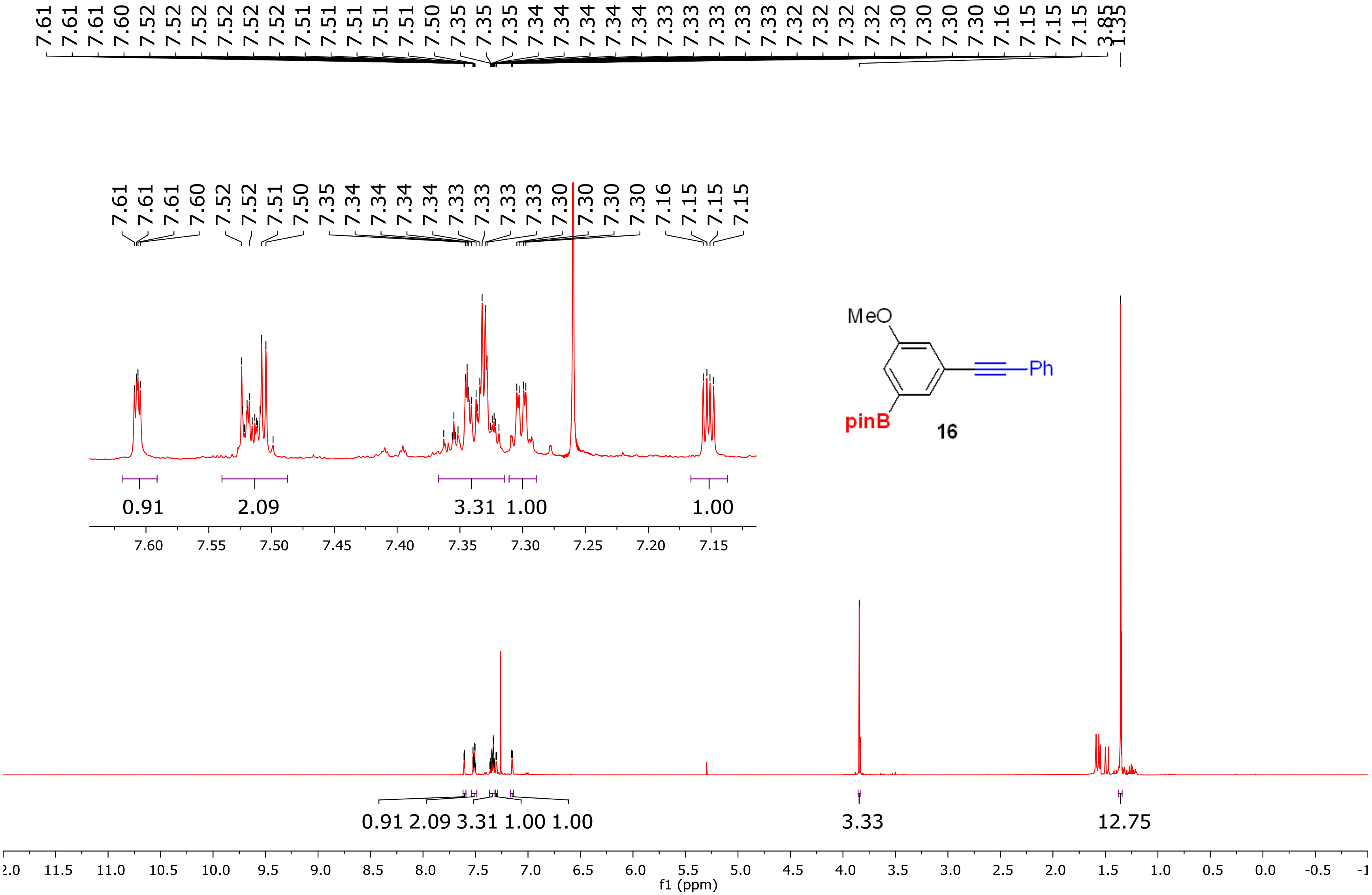

Entry 5: ^{13}C NMR of 16 ($CDCl_3$, 126 MHz)

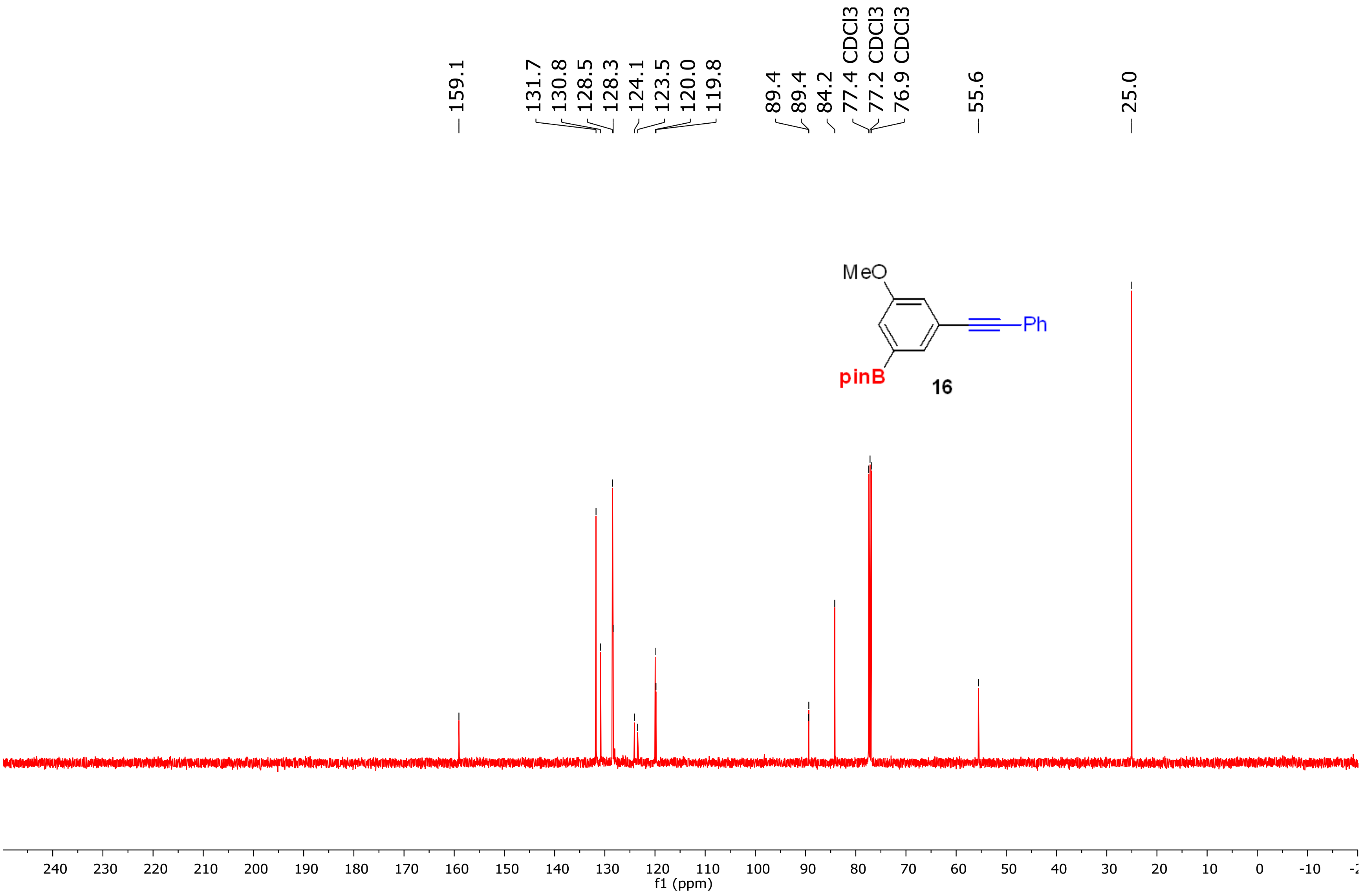

Entry 5: ^{11}B NMR of 16 ($(CD_3)_2CO$, 96 MHz)

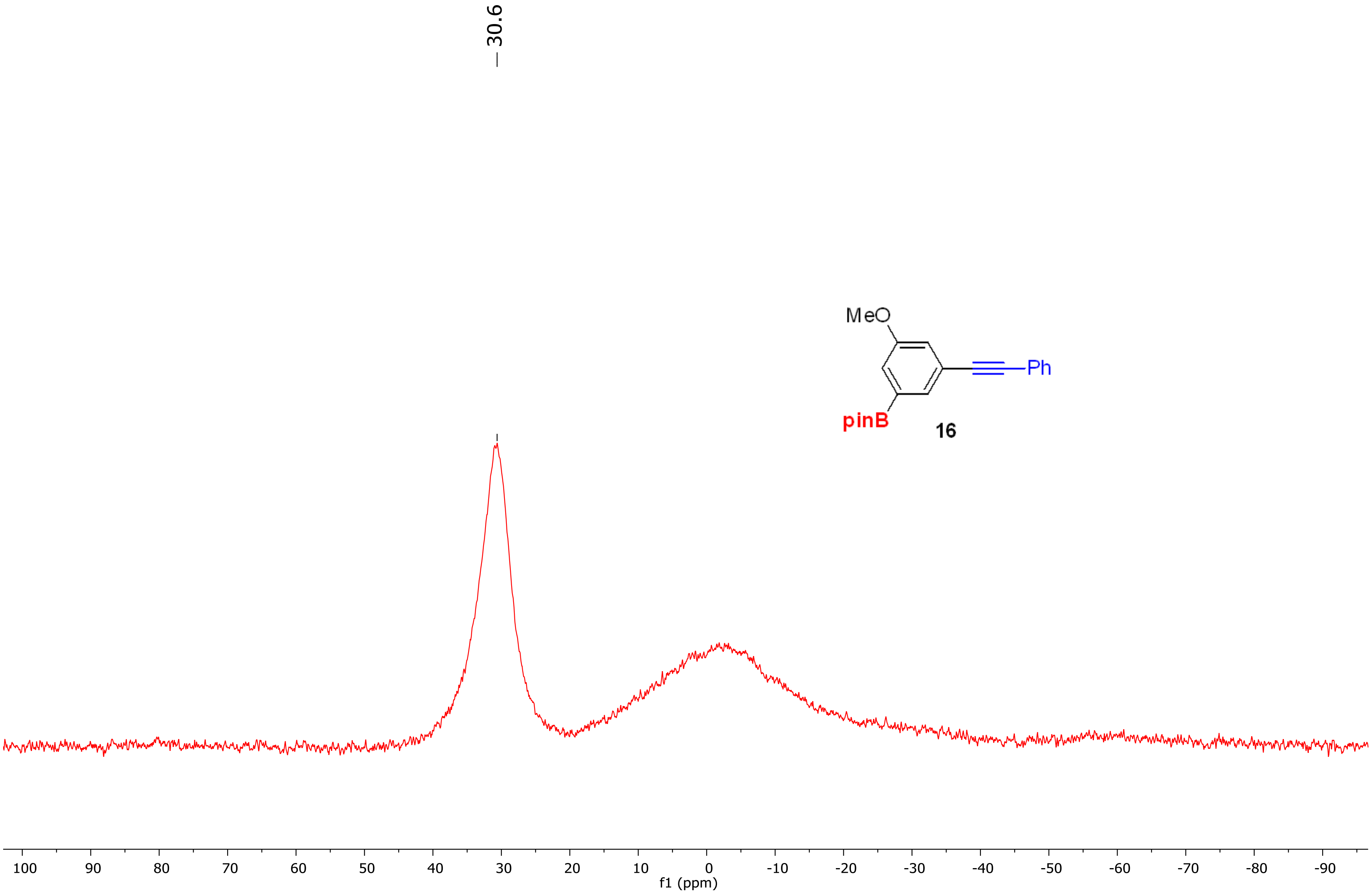

Entry 6: ^1H NMR of 17 (C_6D_6, 300 MHz)

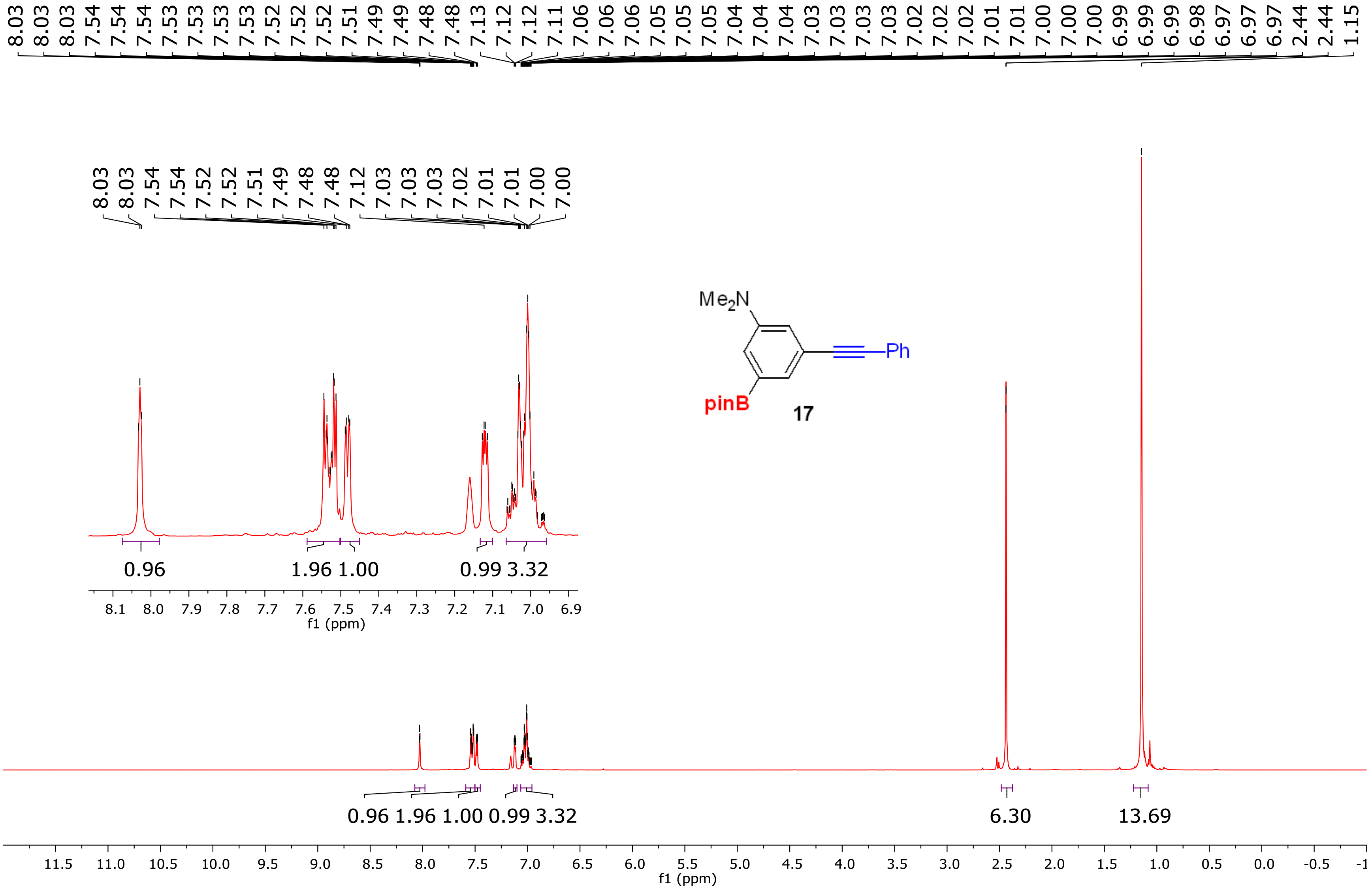

Entry 6: ^{13}C NMR of 17 (C_6D_6, 75 MHz)

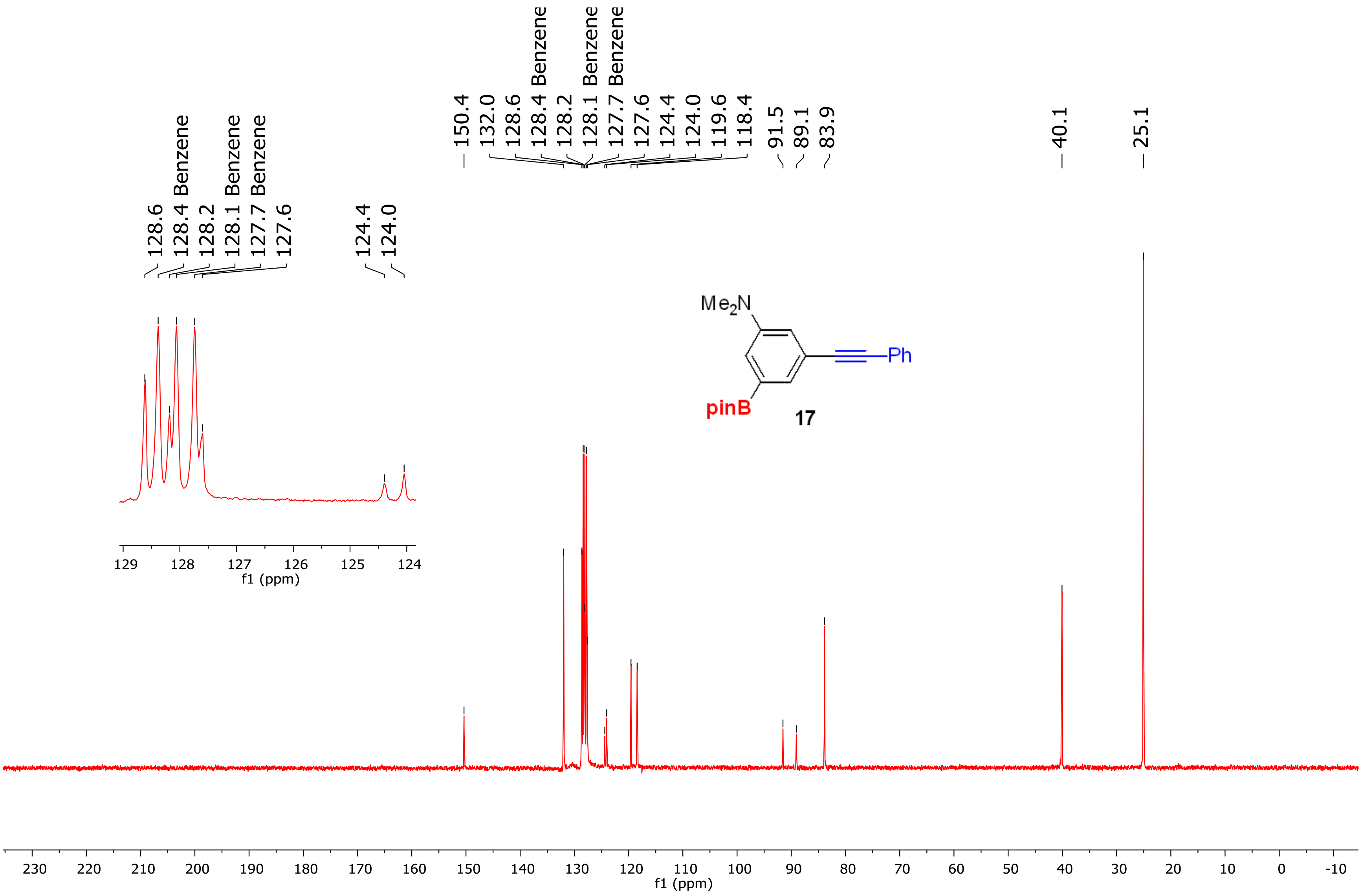

Entry 6: ^{11}B NMR of 17 (C_6D_6, 96 MHz)

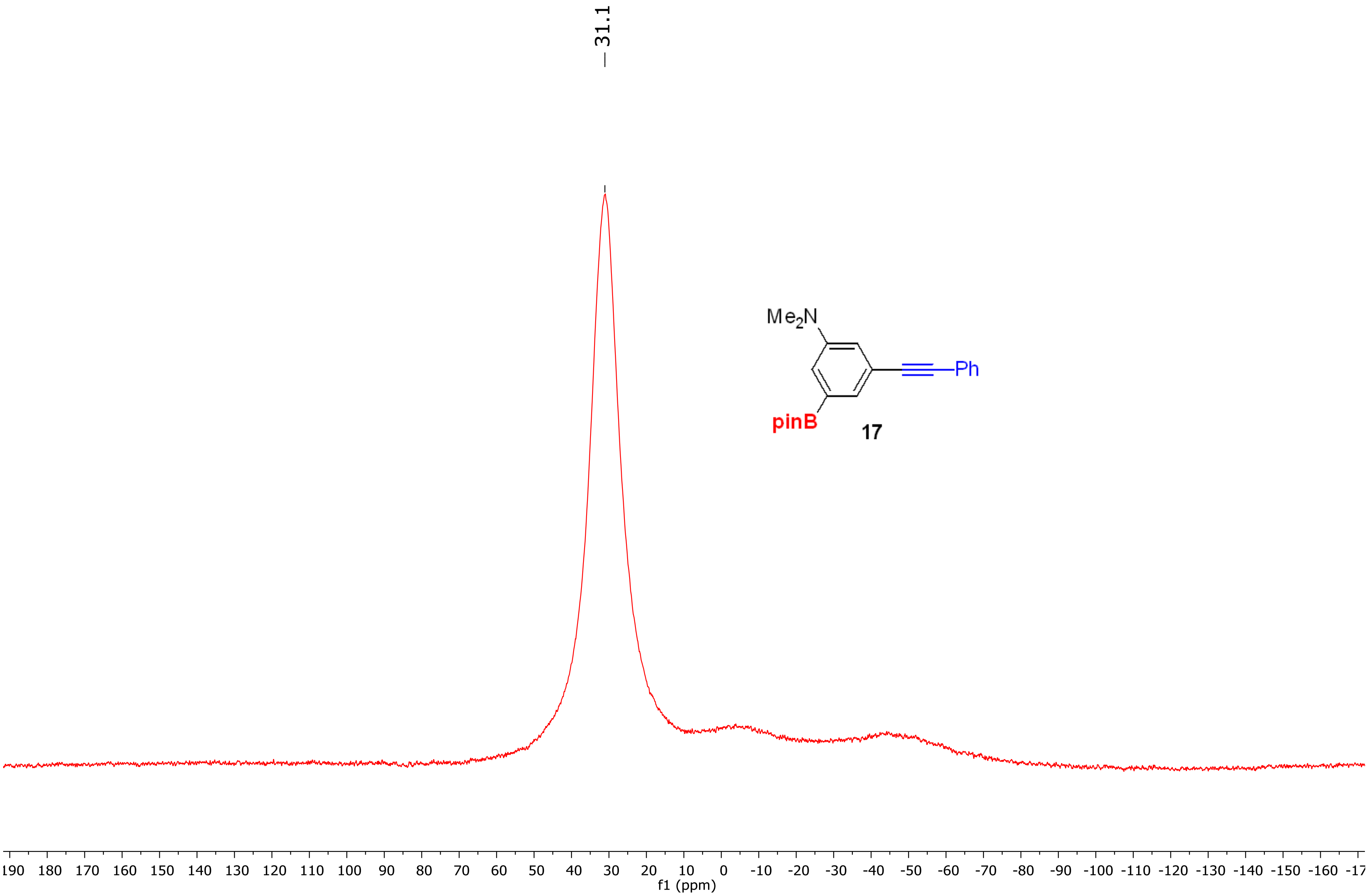

Entry 7: ^{1}H NMR of 18 ($CDCl_3$, 300 MHz)

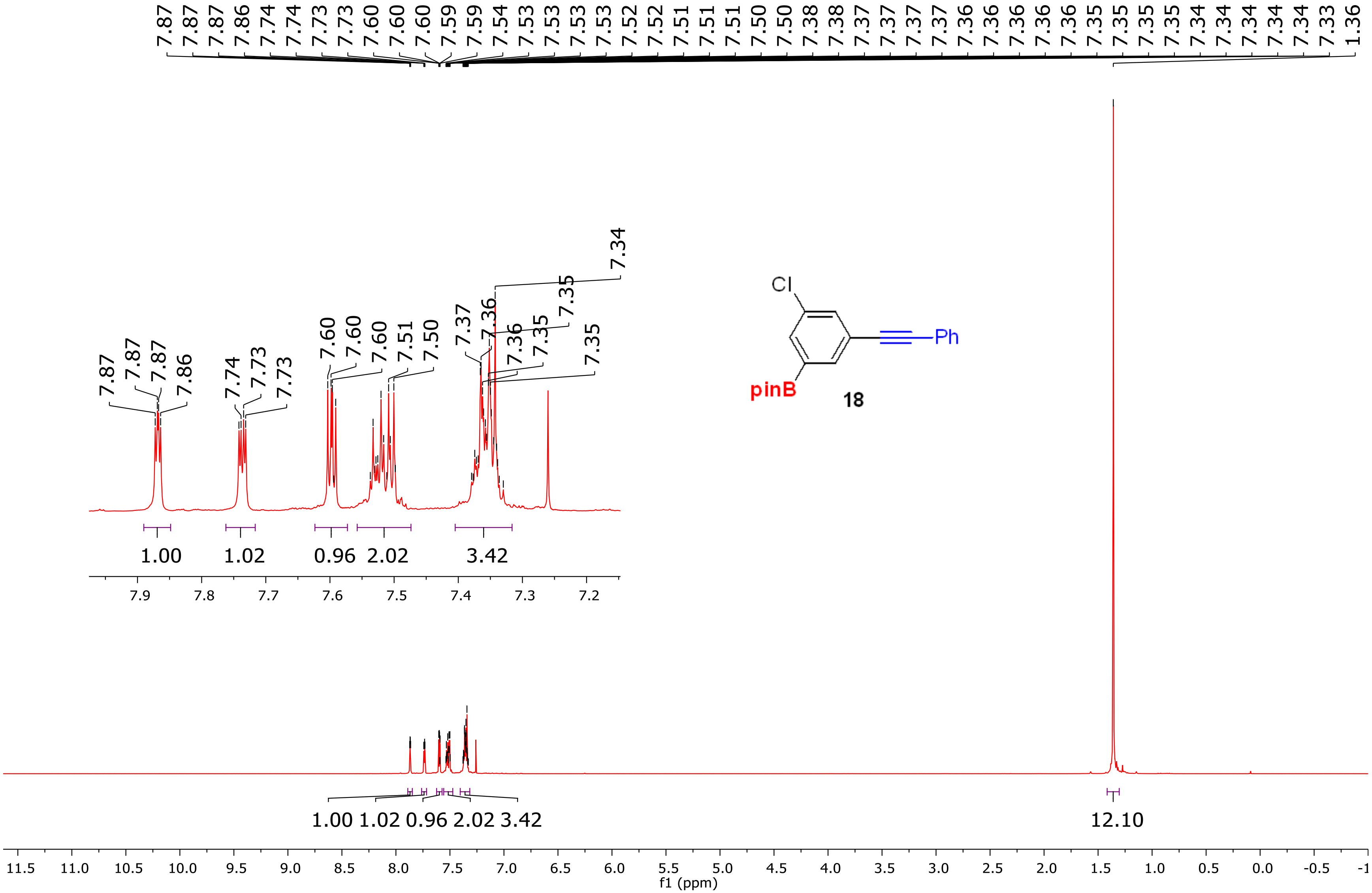

Entry 7: ^{13}C NMR of 18 ($CDCl_3$, 75 MHz)

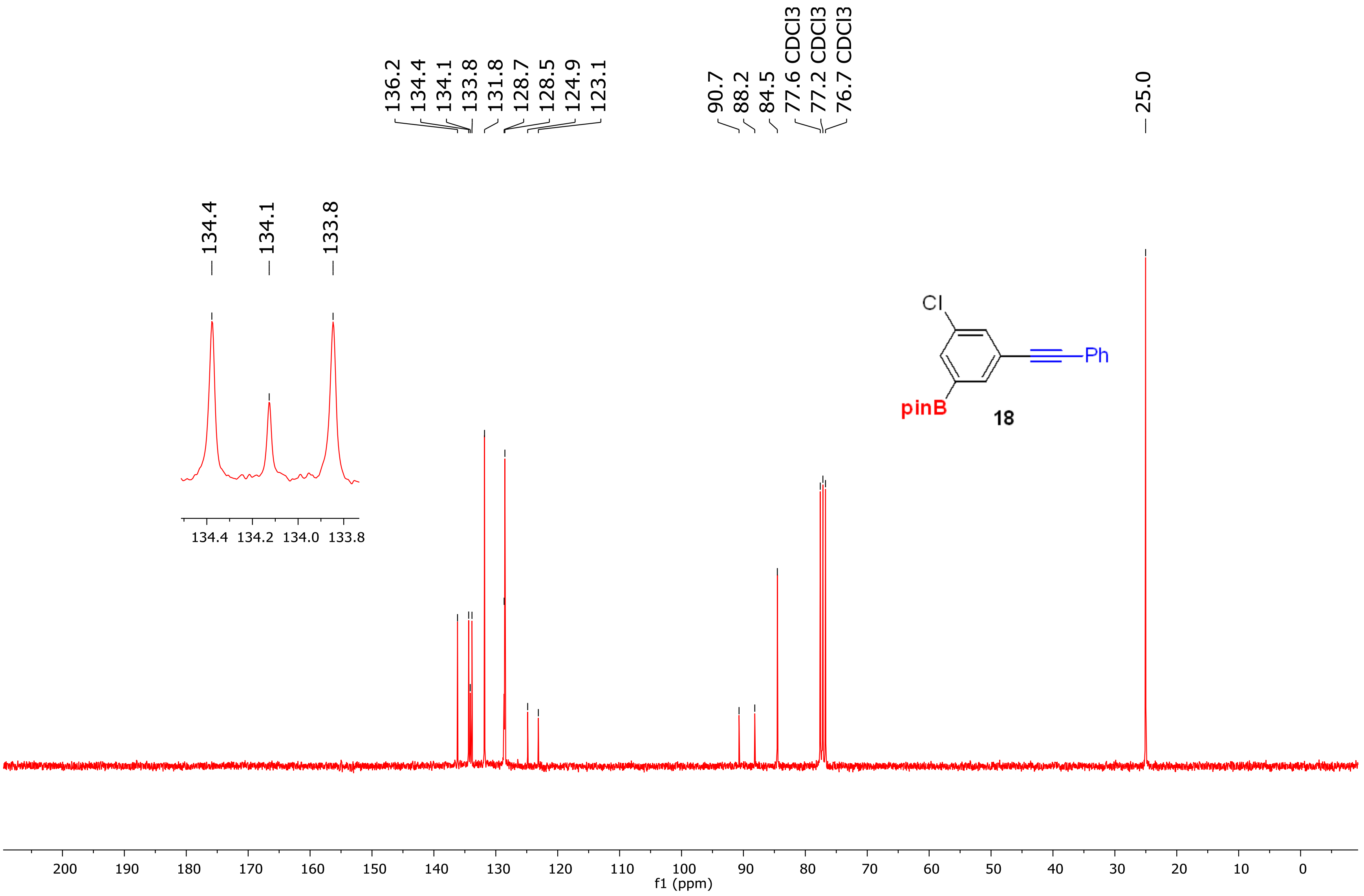

Entry 7: ^{11}B NMR of 18 ($CDCl_3$, 160 MHz)

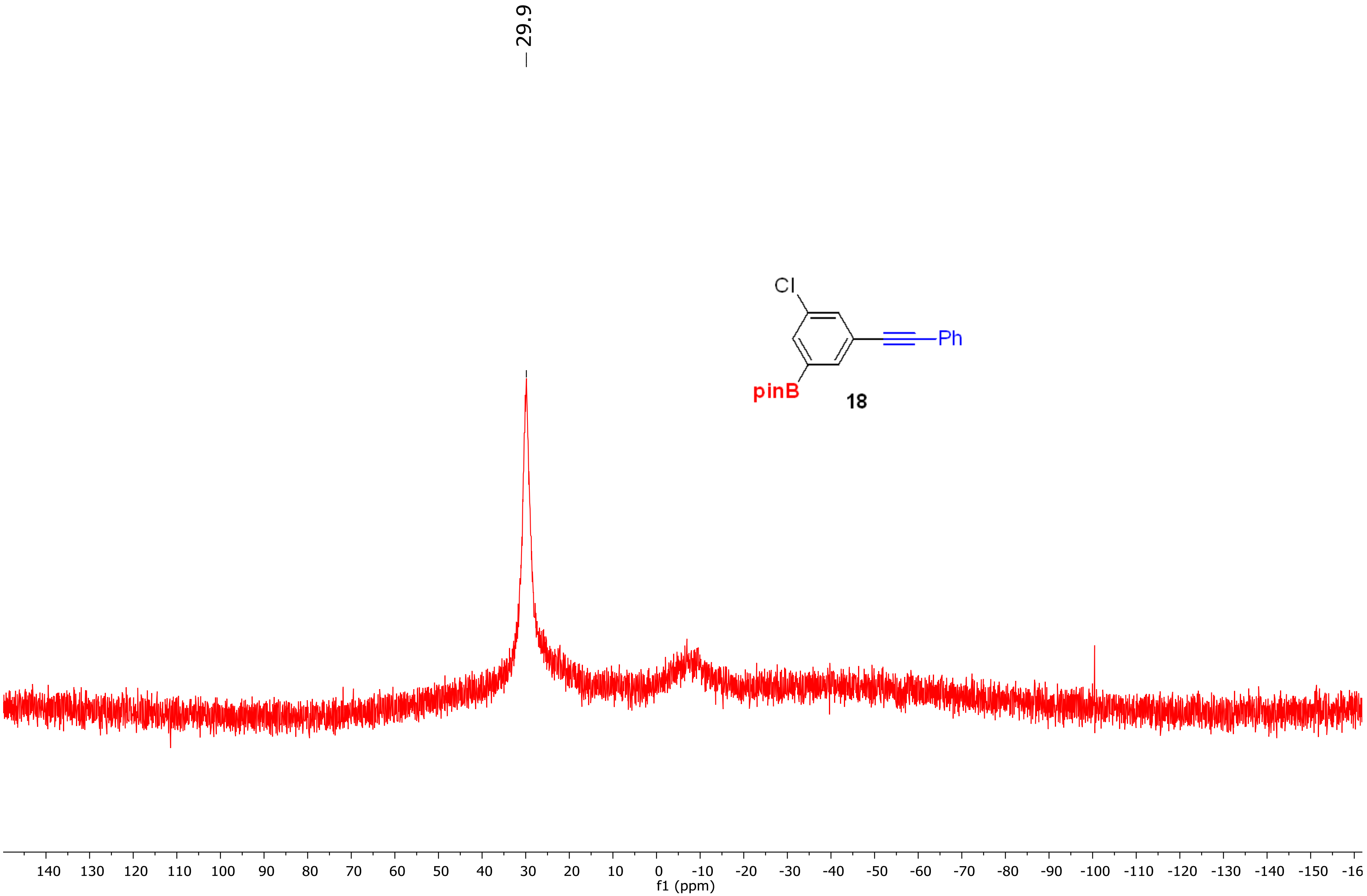

18

Entry 8: ^{1}H NMR of 19 ($CDCl_3$, 300 MHz)

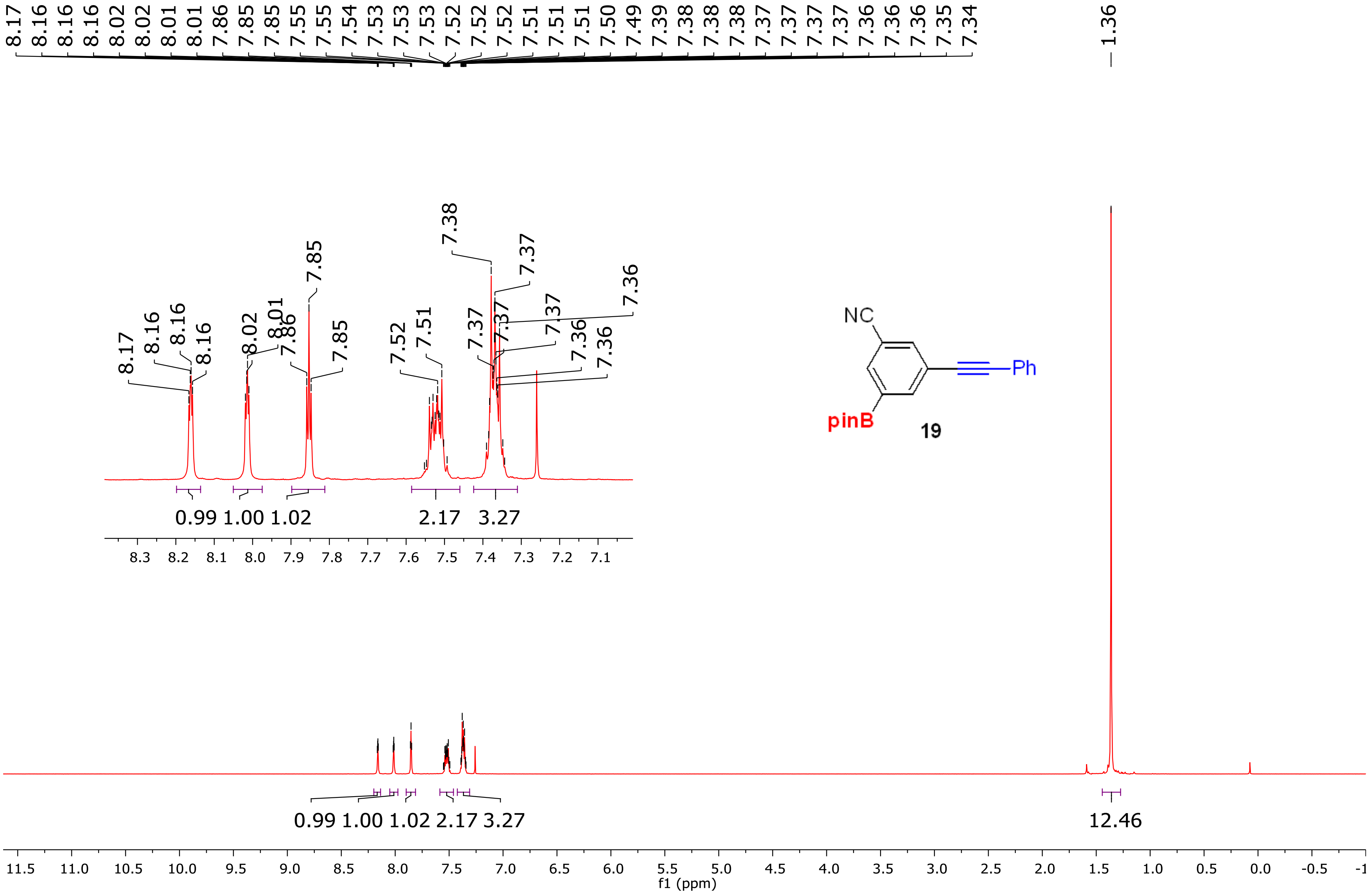

Entry 8: ^{13}C NMR of 19 ($CDCl_3$, 75 MHz)

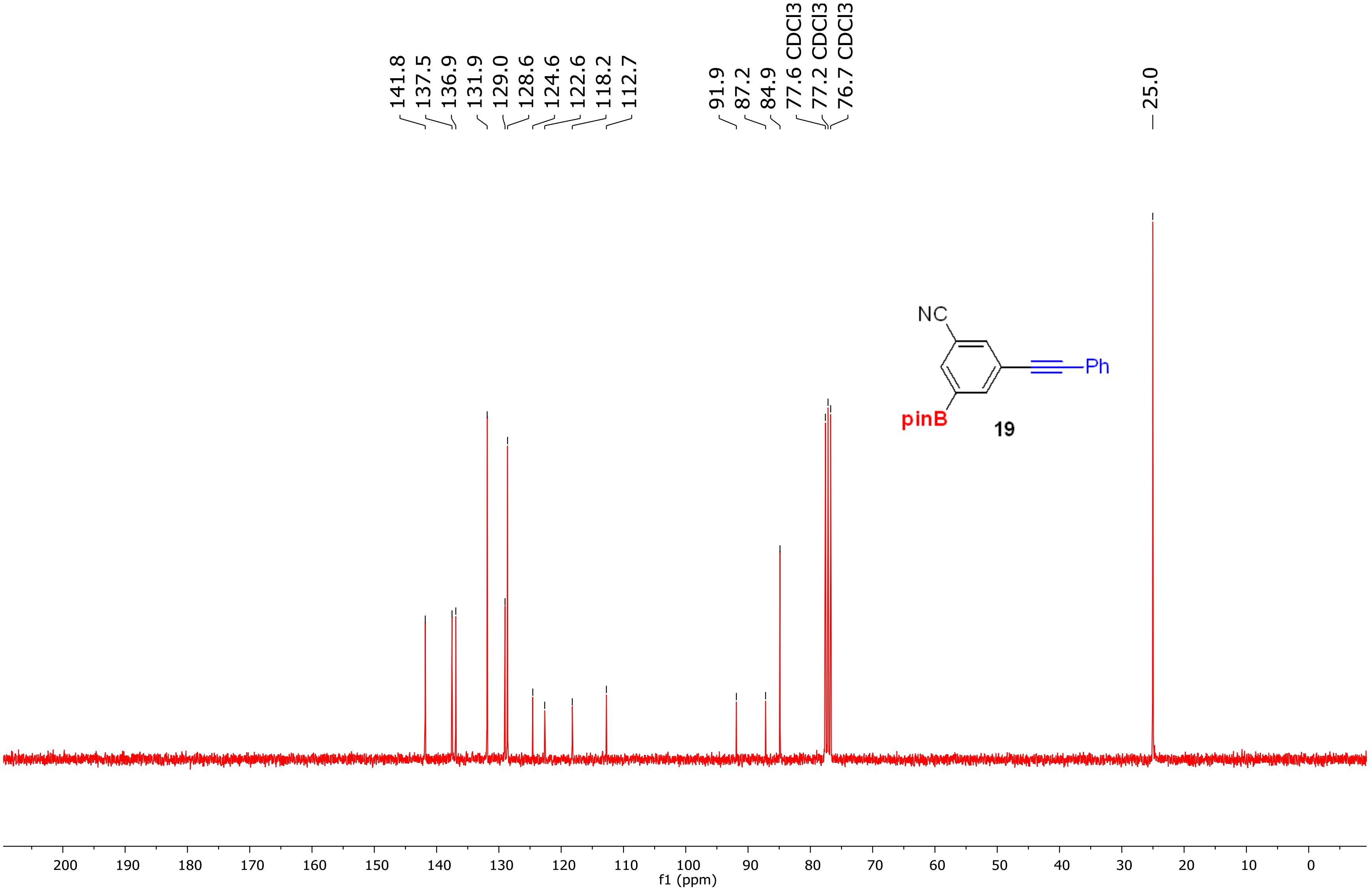

Entry 8: ^{11}B NMR of 19 ($CDCl_3$, 160 MHz)

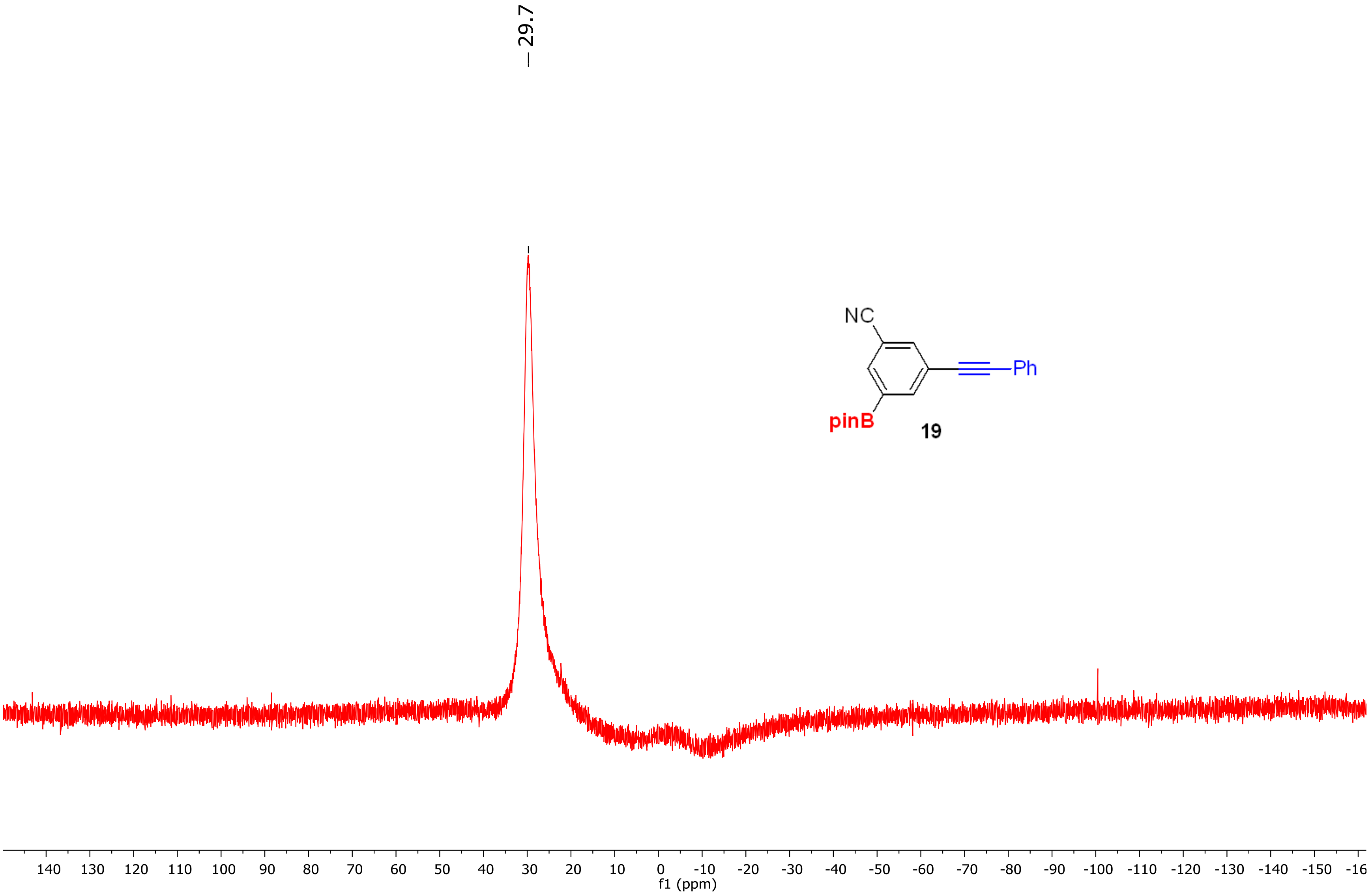

Entry 9: ^{1}H NMR of 20 ($CDCl_3$, 500 MHz)

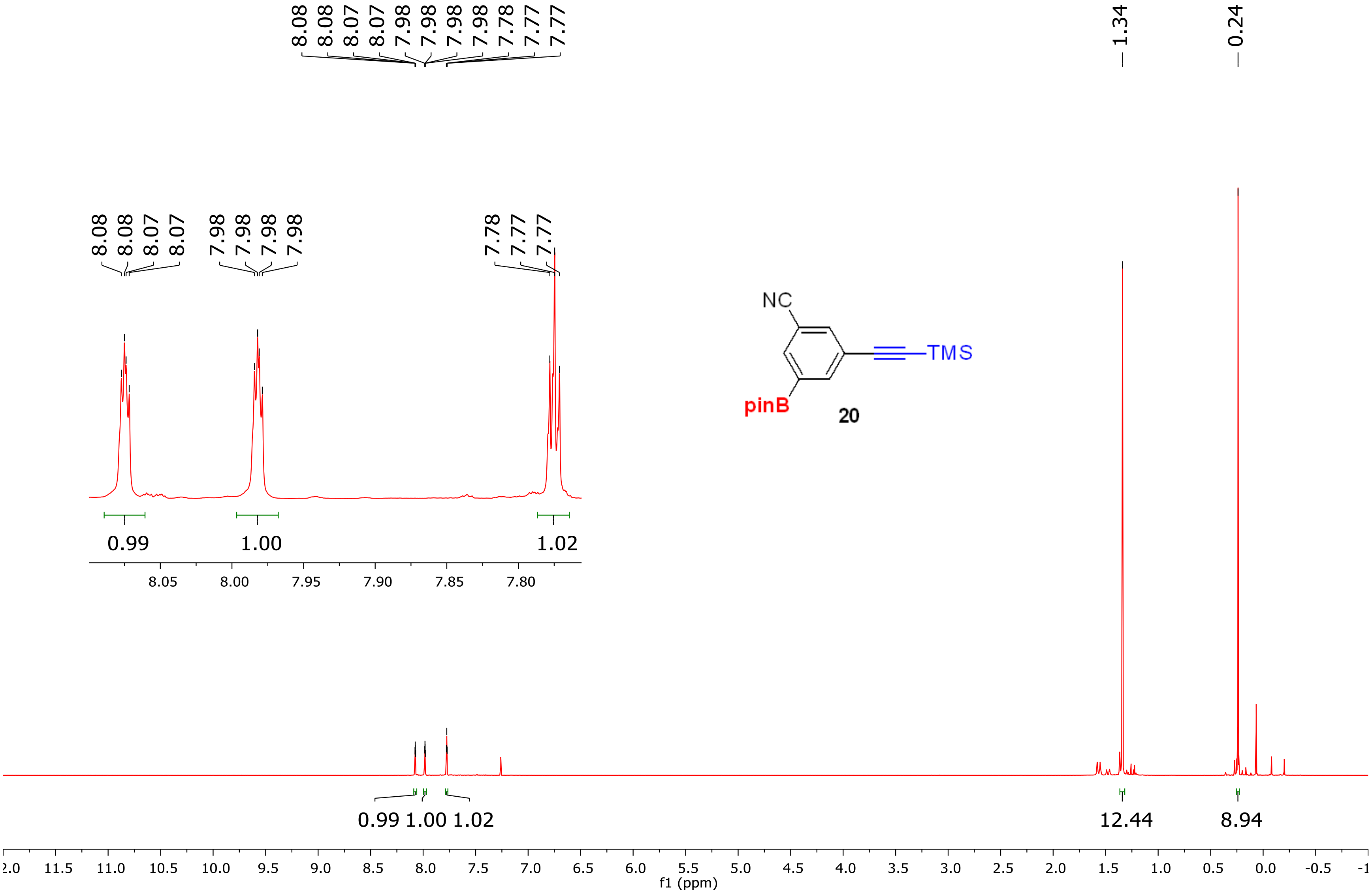

Entry 9: ^{13}C NMR of 20 ($CDCl_3$, 126 MHz)

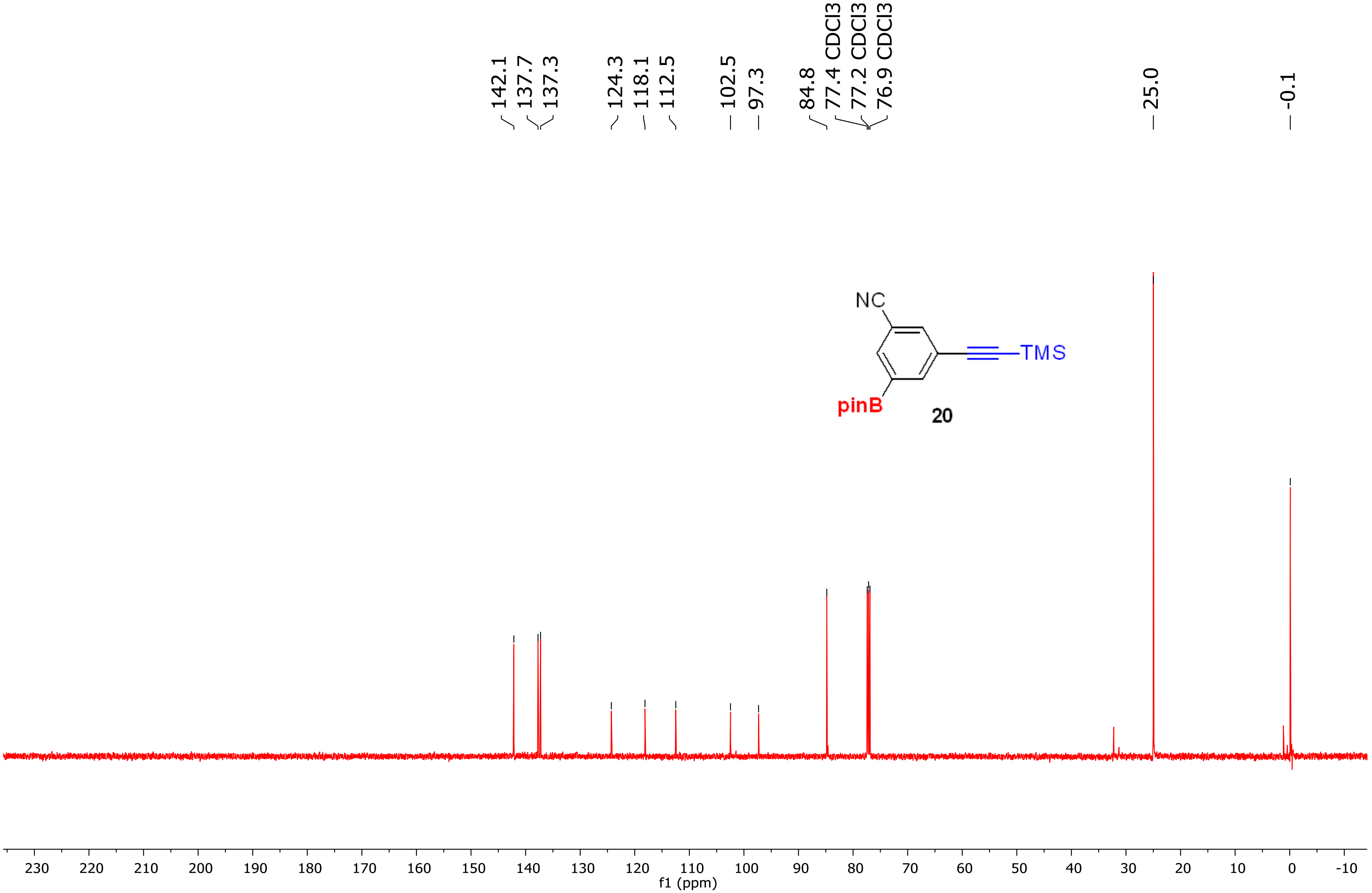

Entry 9: ¹¹B NMR of 20 (CDCl₃, 160 MHz)

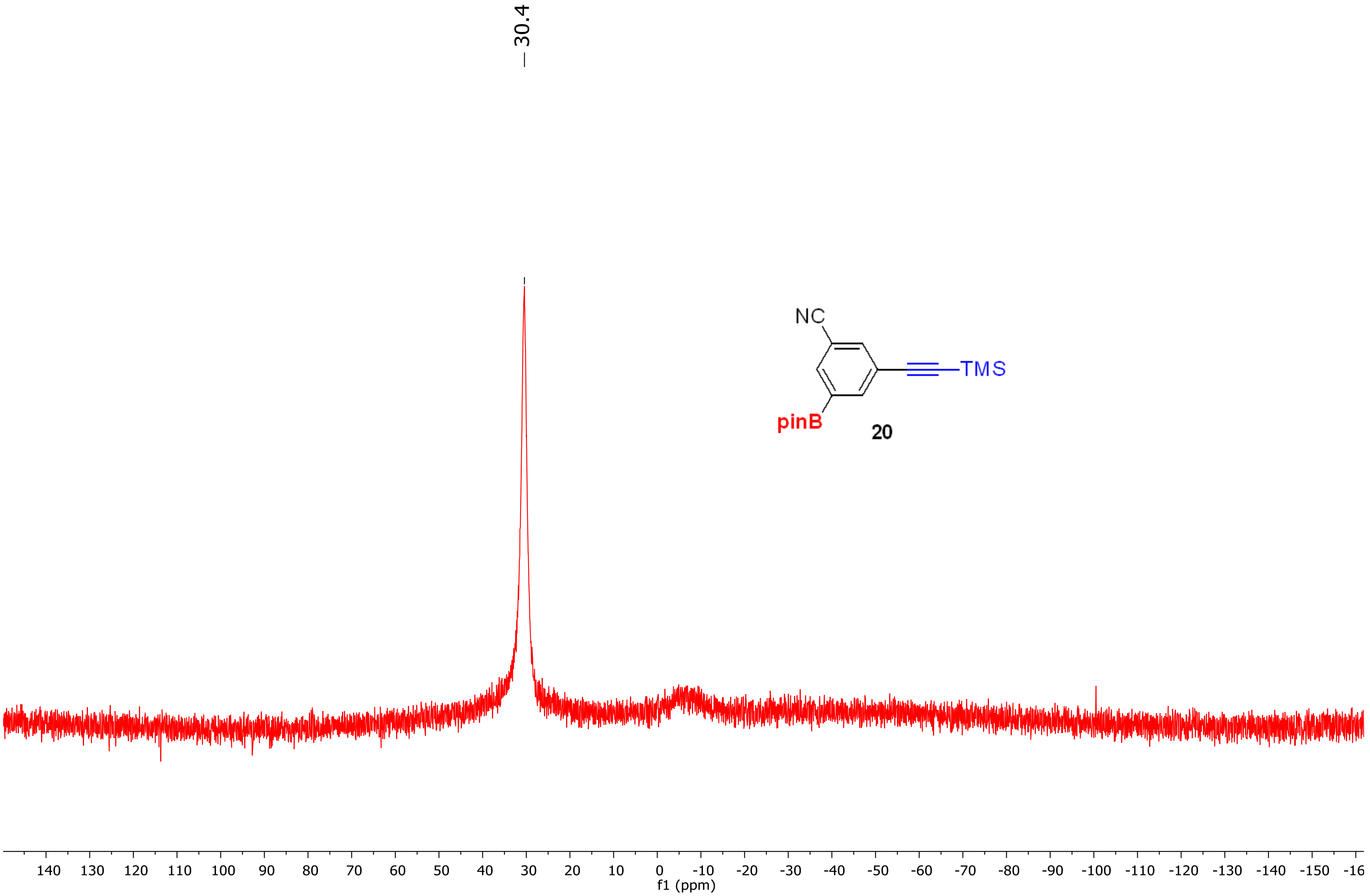

Entry 10: ¹H NMR of 21 ($CDCl_3$, 500 MHz)

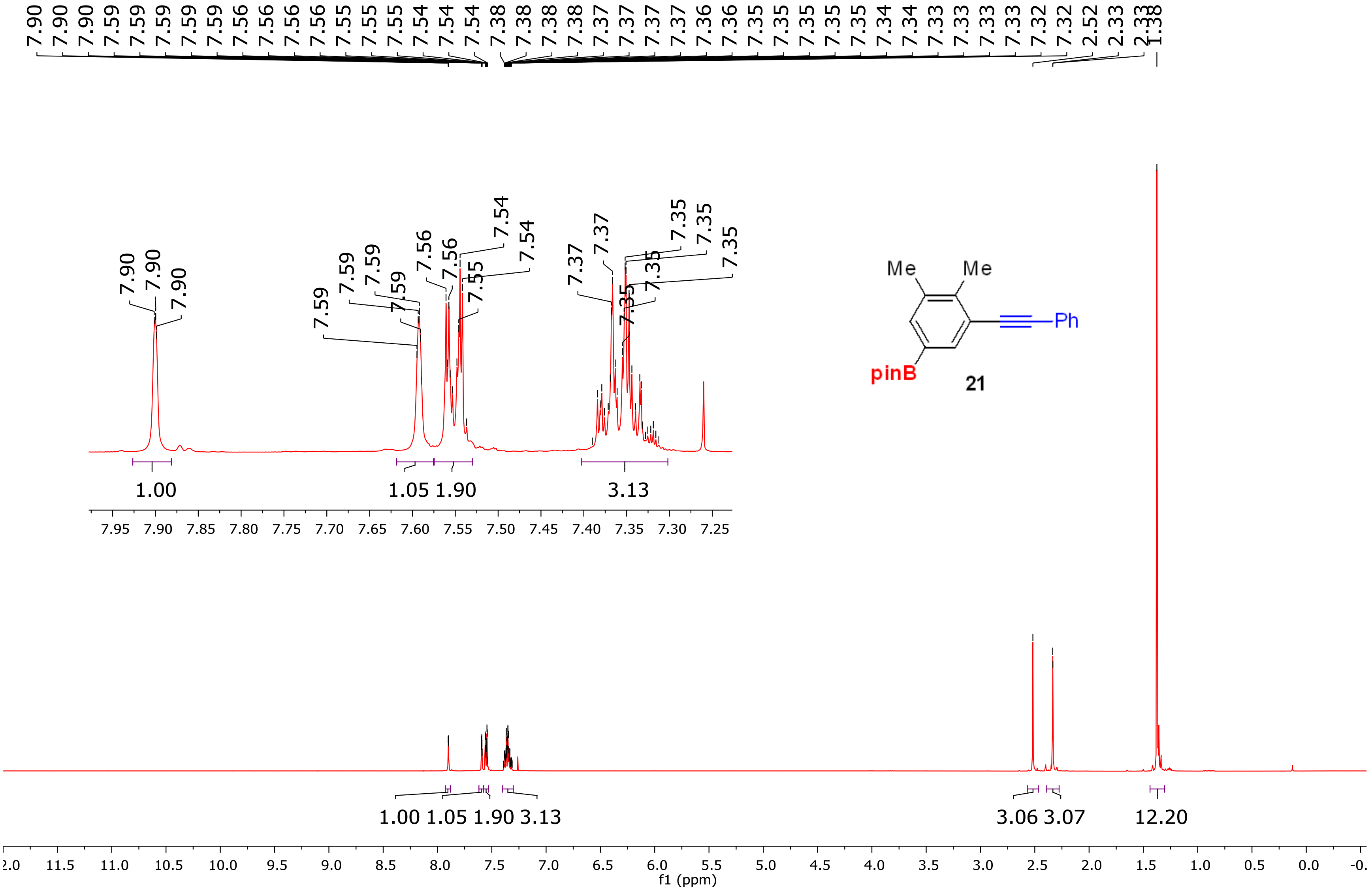

Entry 10: ^{13}C NMR of 21 (CDCl$_3$, 126 MHz)

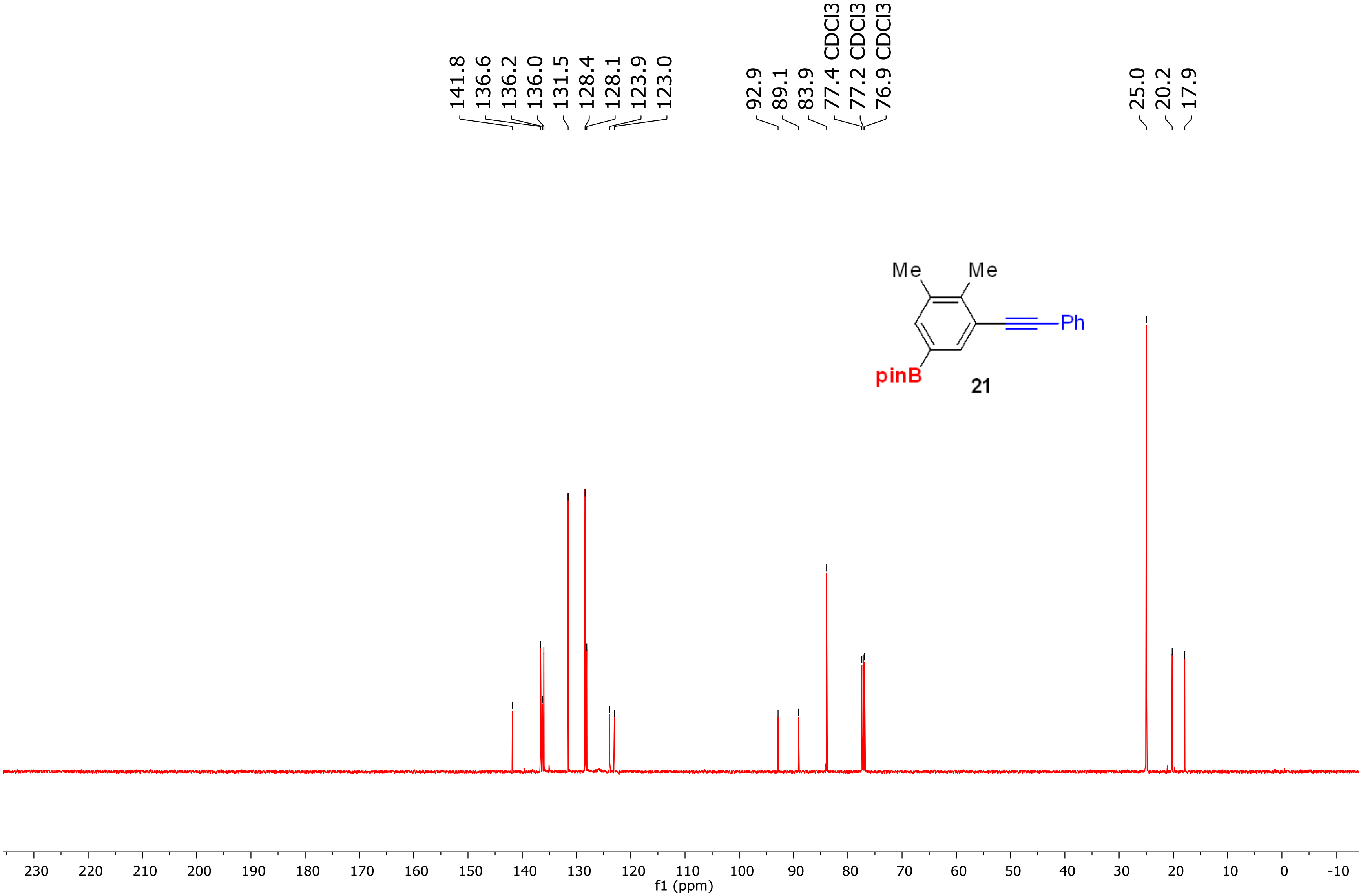

Entry 10: ^{11}B NMR of 21 ($CDCl_3$, 160 MHz)

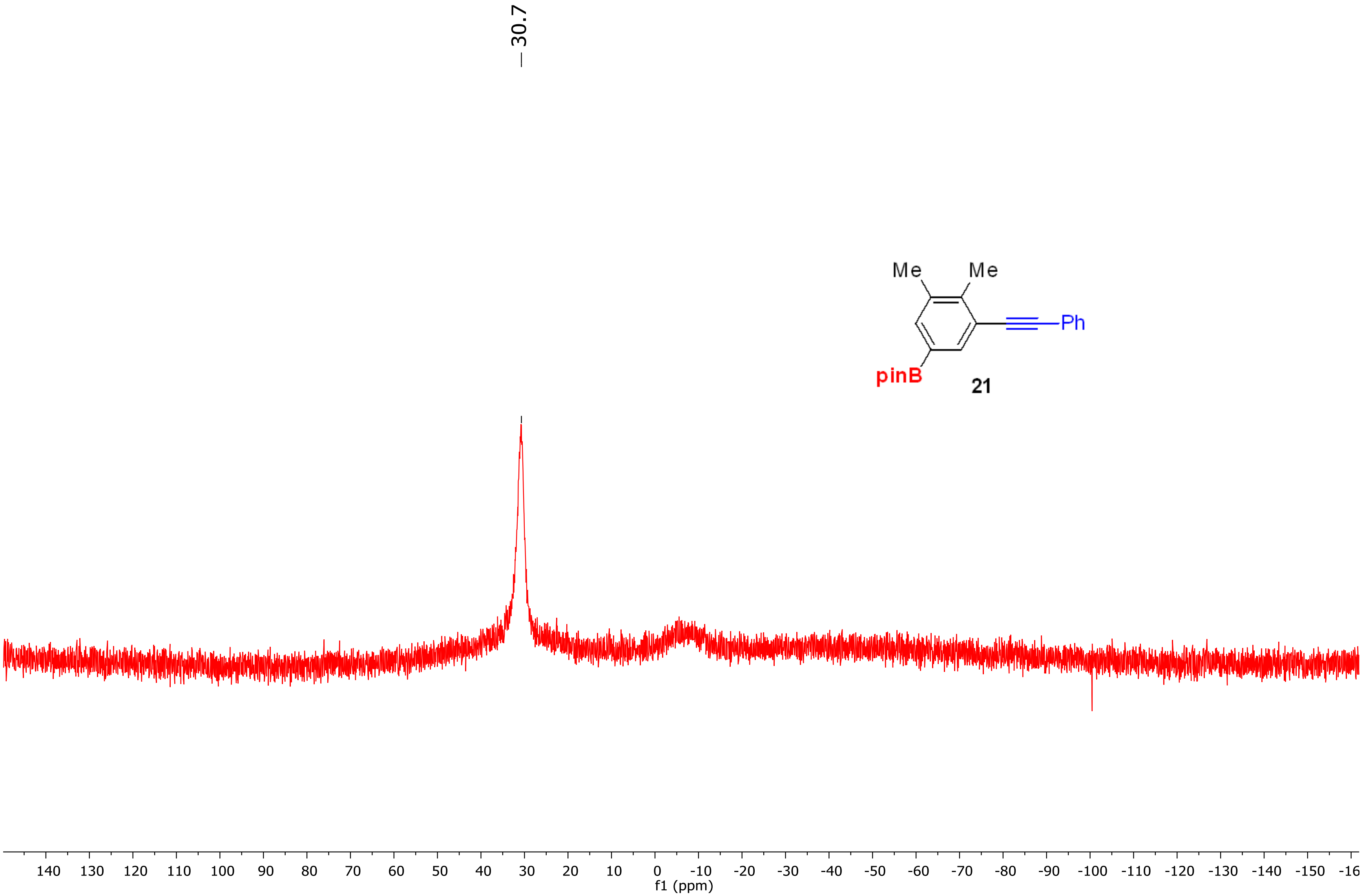

Entry 11: ^{1}H NMR of 22 ($CDCl_3$, 500 MHz)

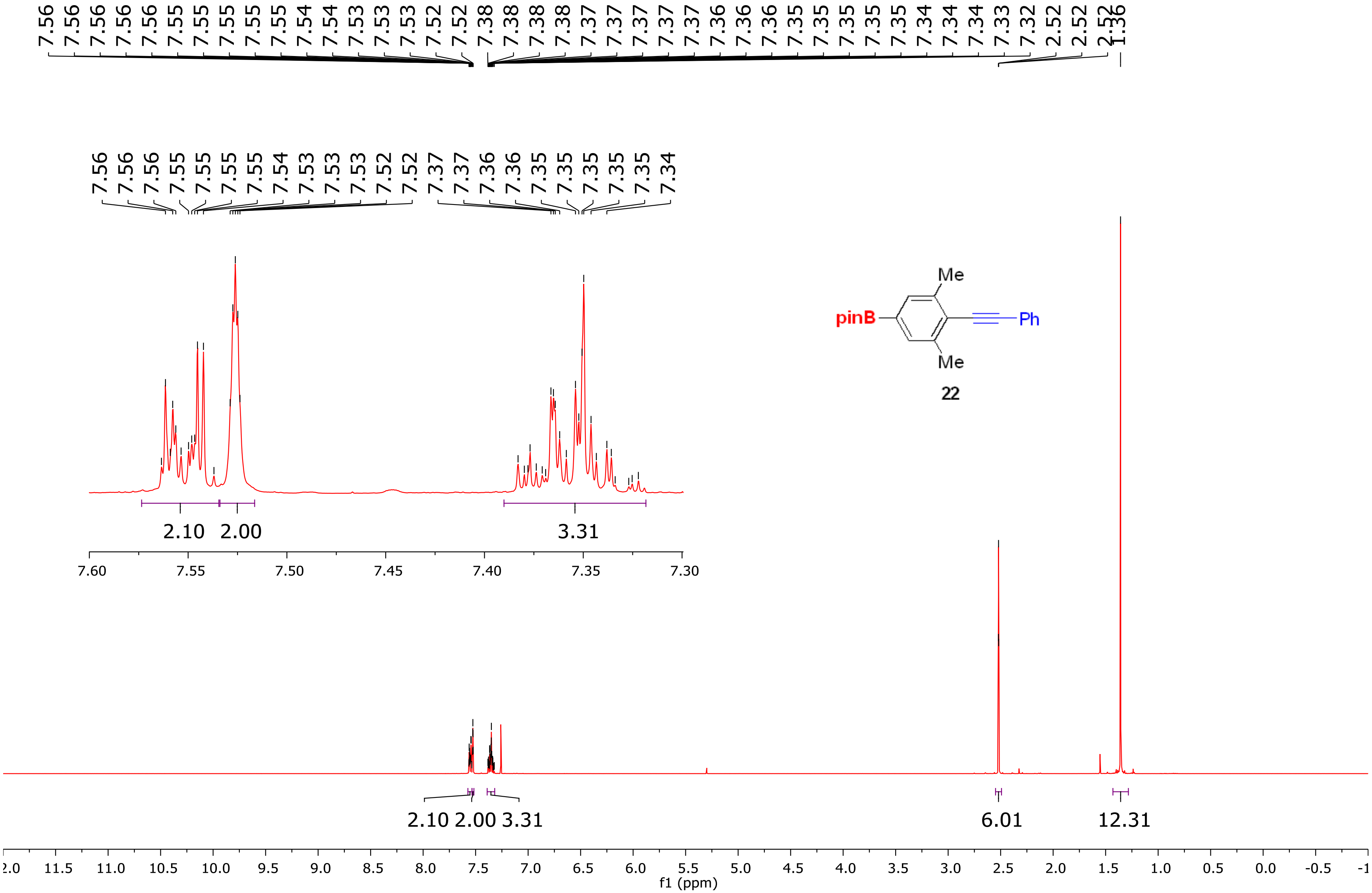

Entry 11: ^{13}C NMR of 22 ($CDCl_3$, 126 MHz)

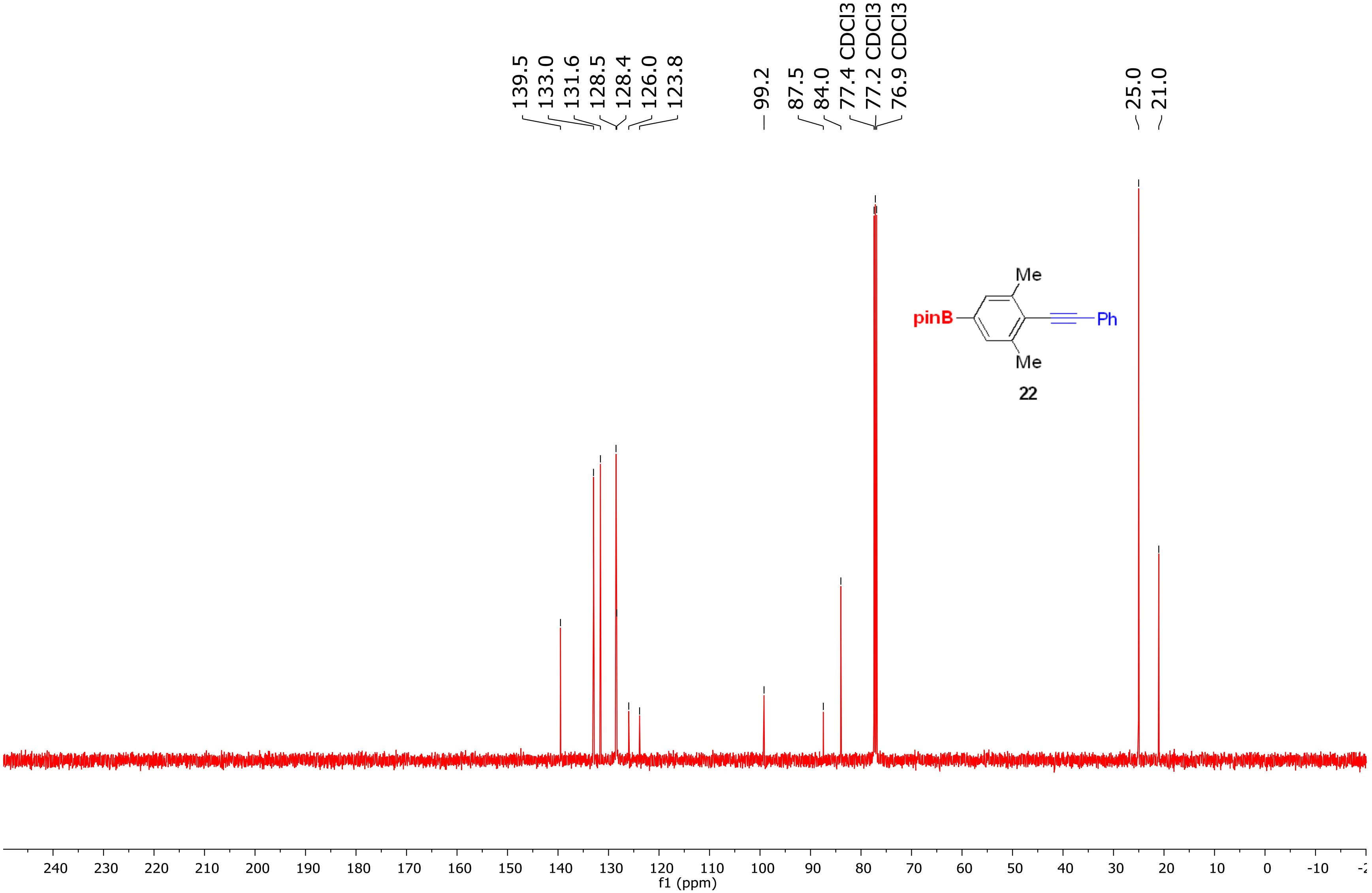

Entry 11: ^{11}B NMR of 22 ($(CD_3)_2CO$, 96 MHz)

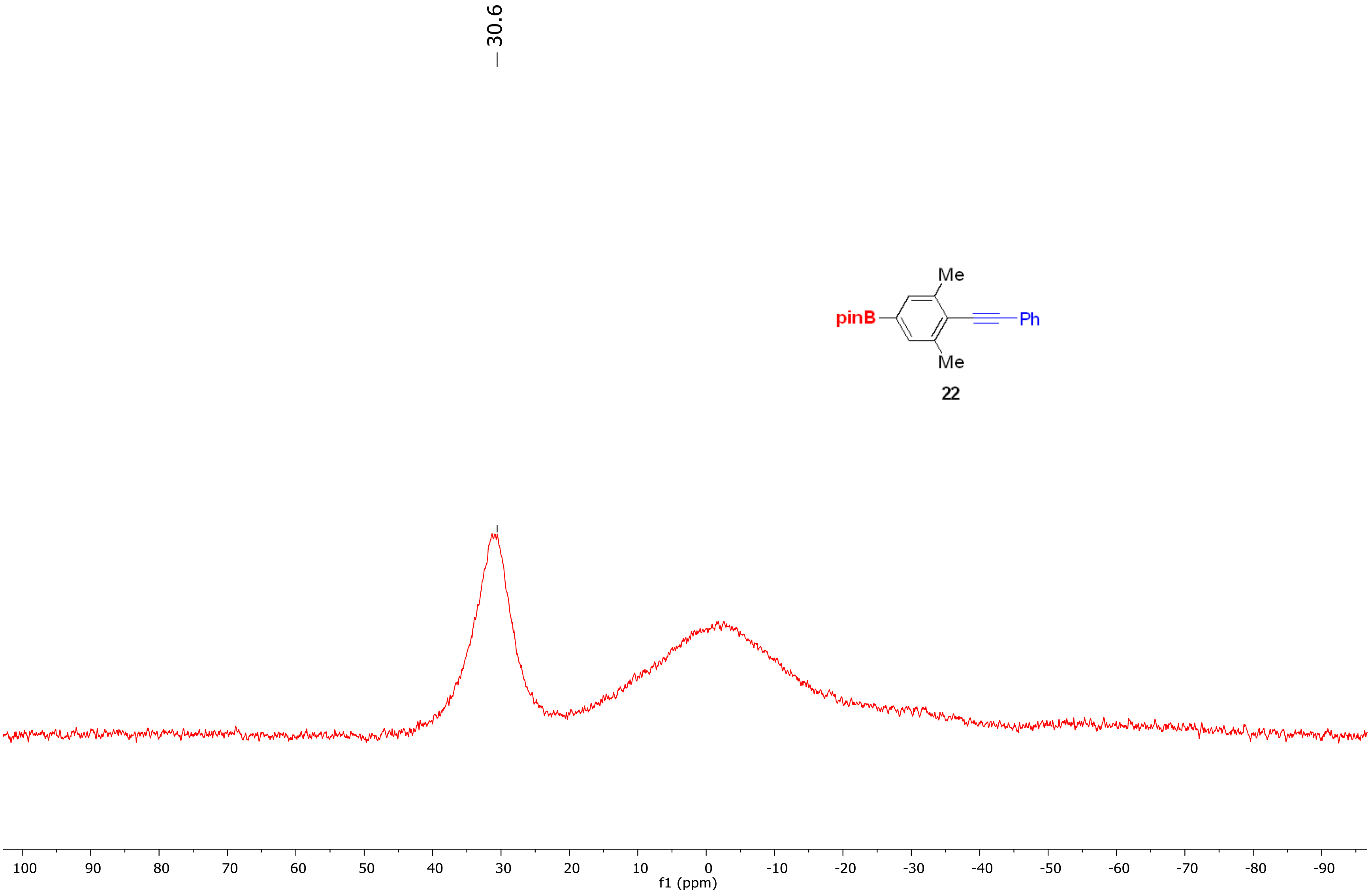

Entry 12: ^{1}H NMR of 23 ($CDCl_3$, 500 MHz)

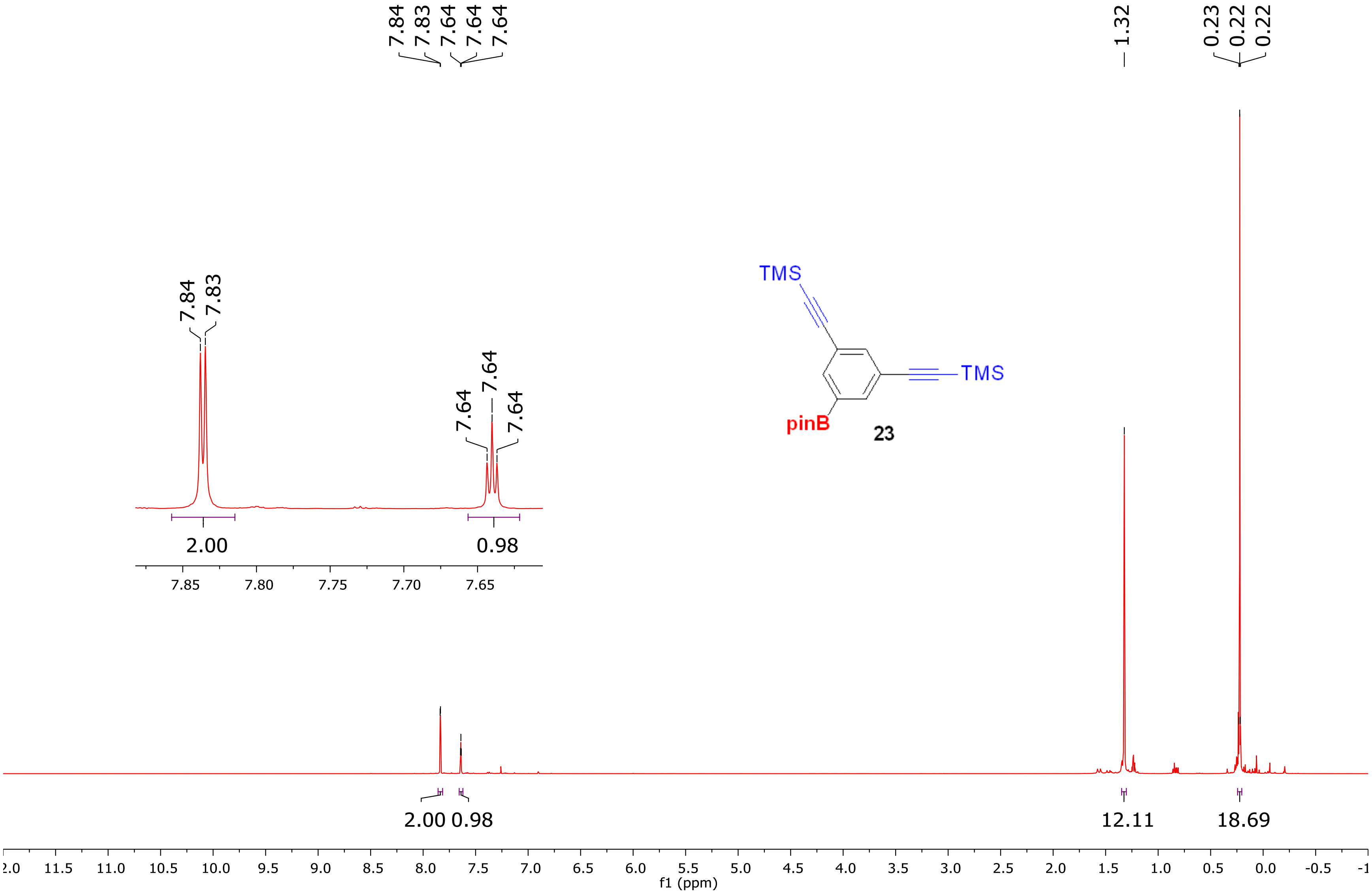

Entry 12: ^{13}C NMR of 23 ($CDCl_3$, 126 MHz)

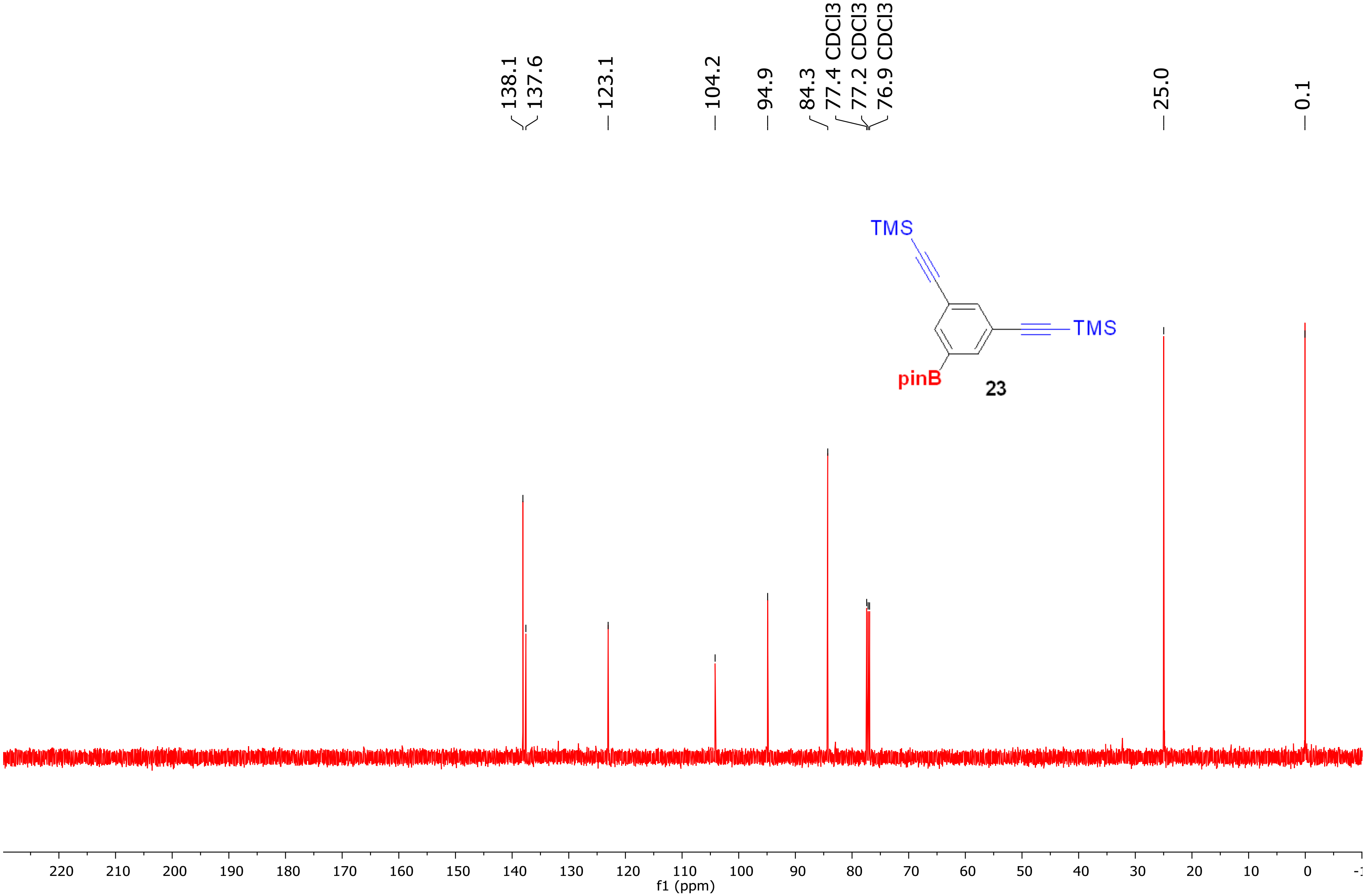

Entry 12: ^{11}B NMR of 23 ($CDCl_3$, 160 MHz)

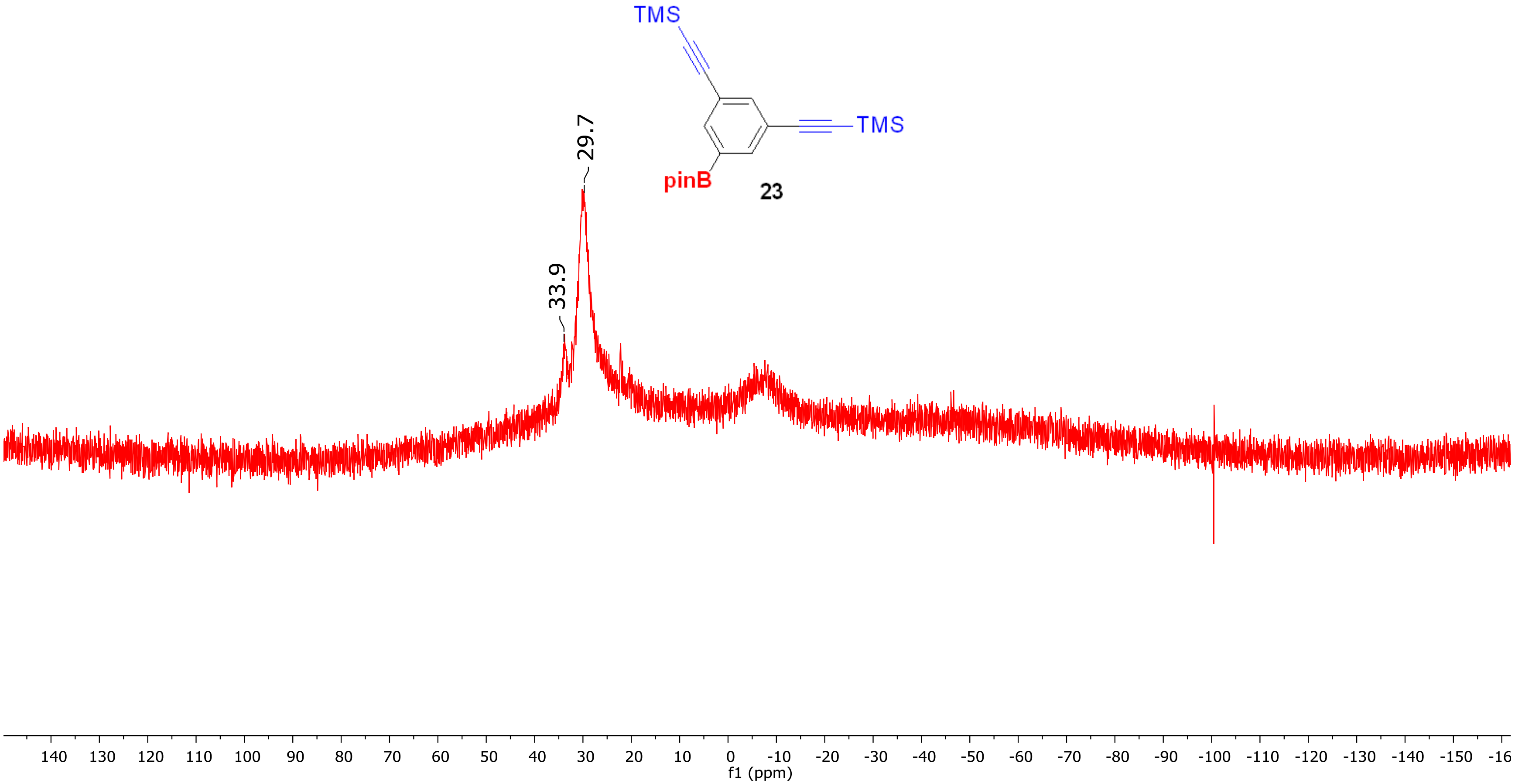

Entry 13: ^{1}H NMR of 24 ($CDCl_3$, 500 MHz)

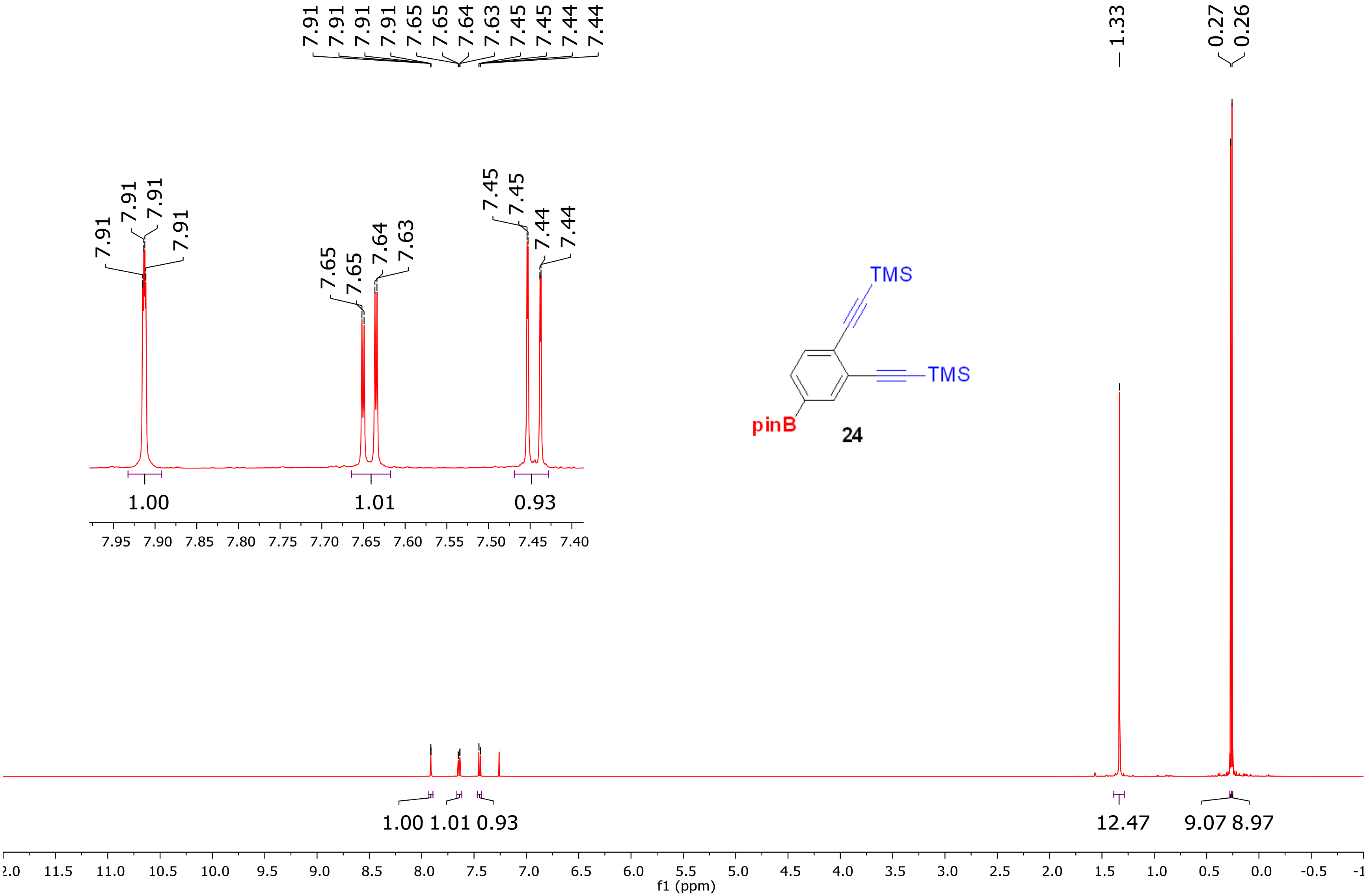

Entry 13: ^{13}C NMR of 24 ($CDCl_3$, 126 MHz)

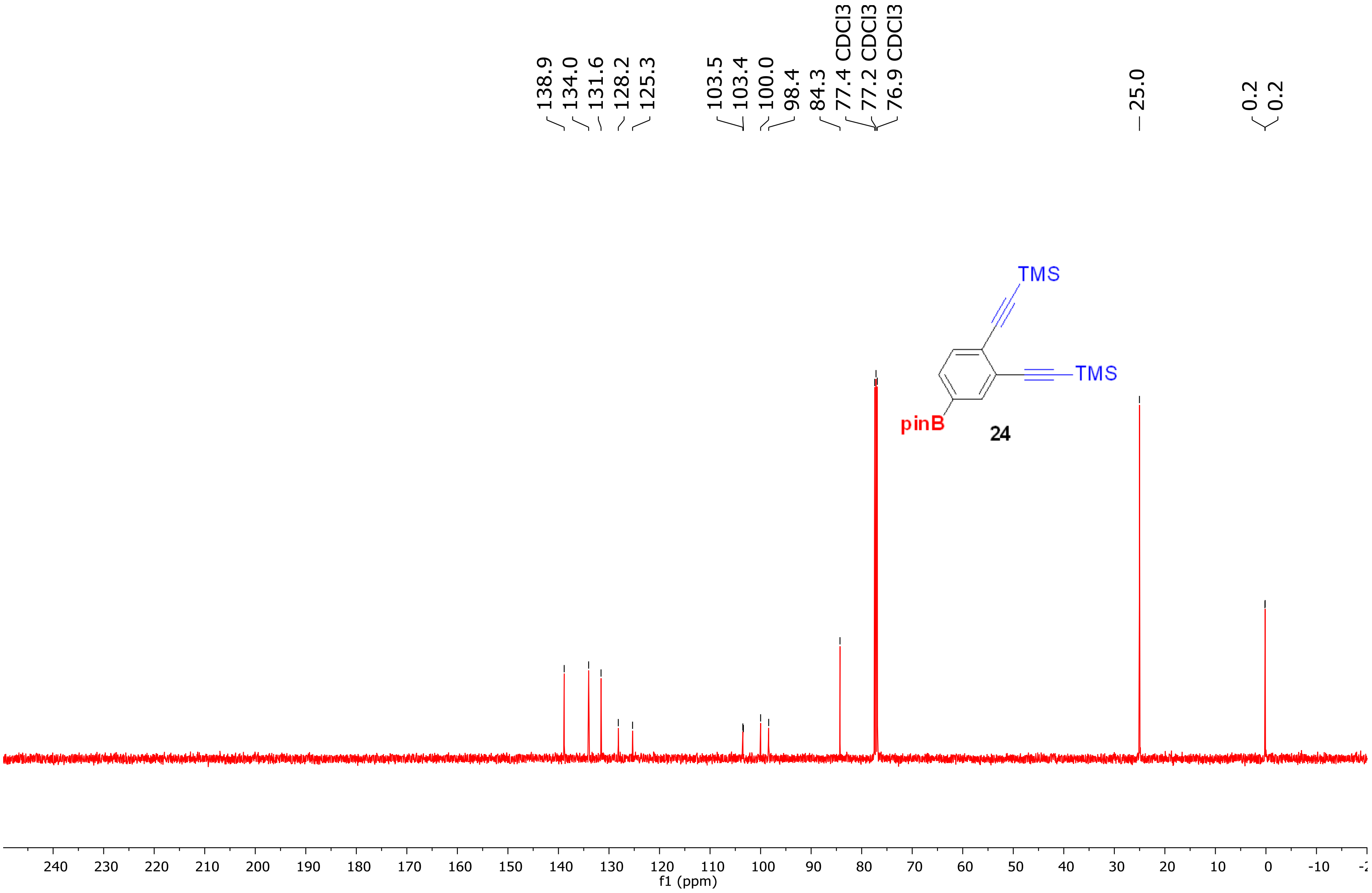

Entry 13: ^{11}B NMR of 24 ($(CD_3)_2CO$, 96 MHz)

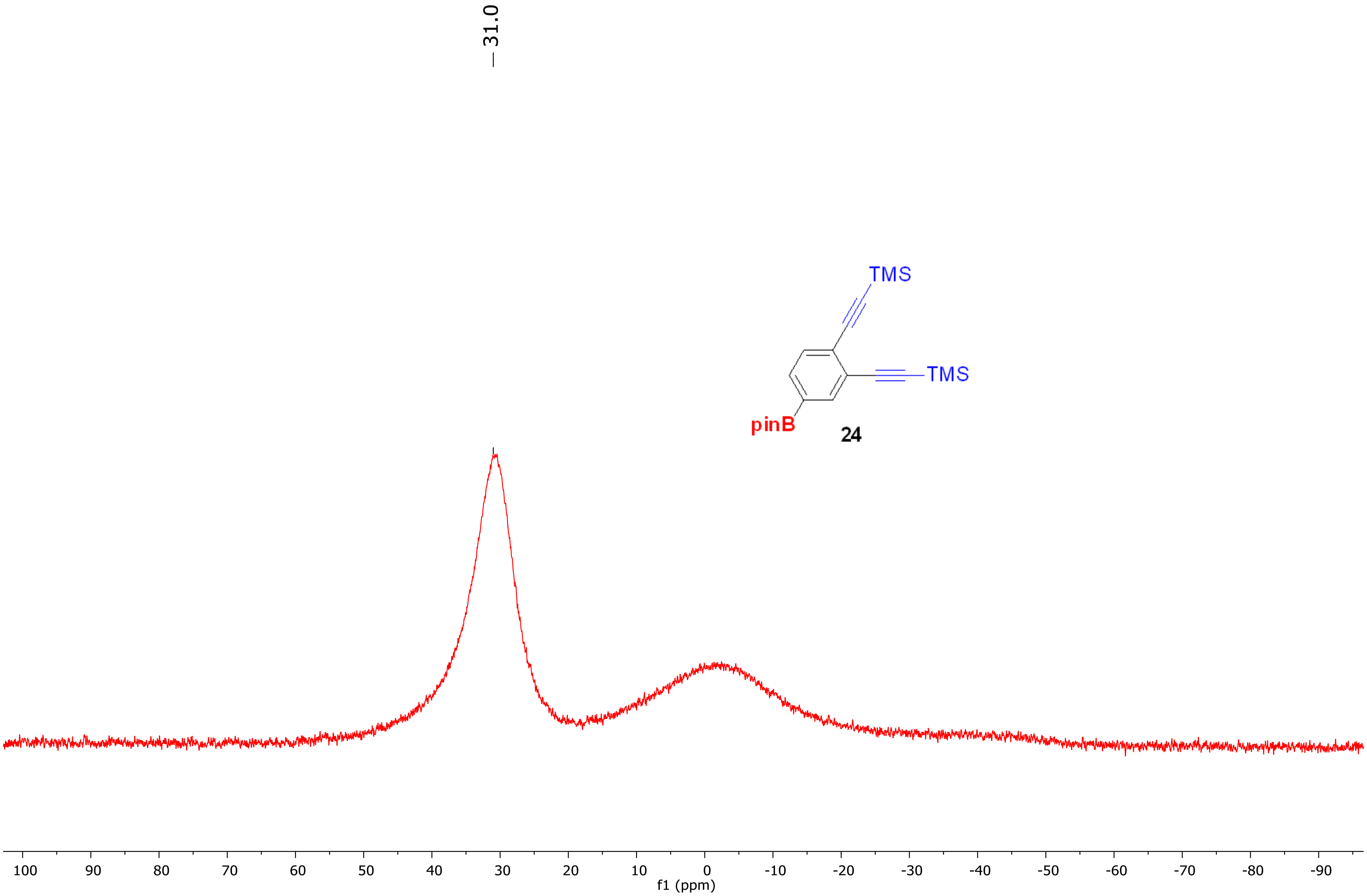

Entry 14: ^{1}H NMR of 25 ($CDCl_3$, 500 MHz)

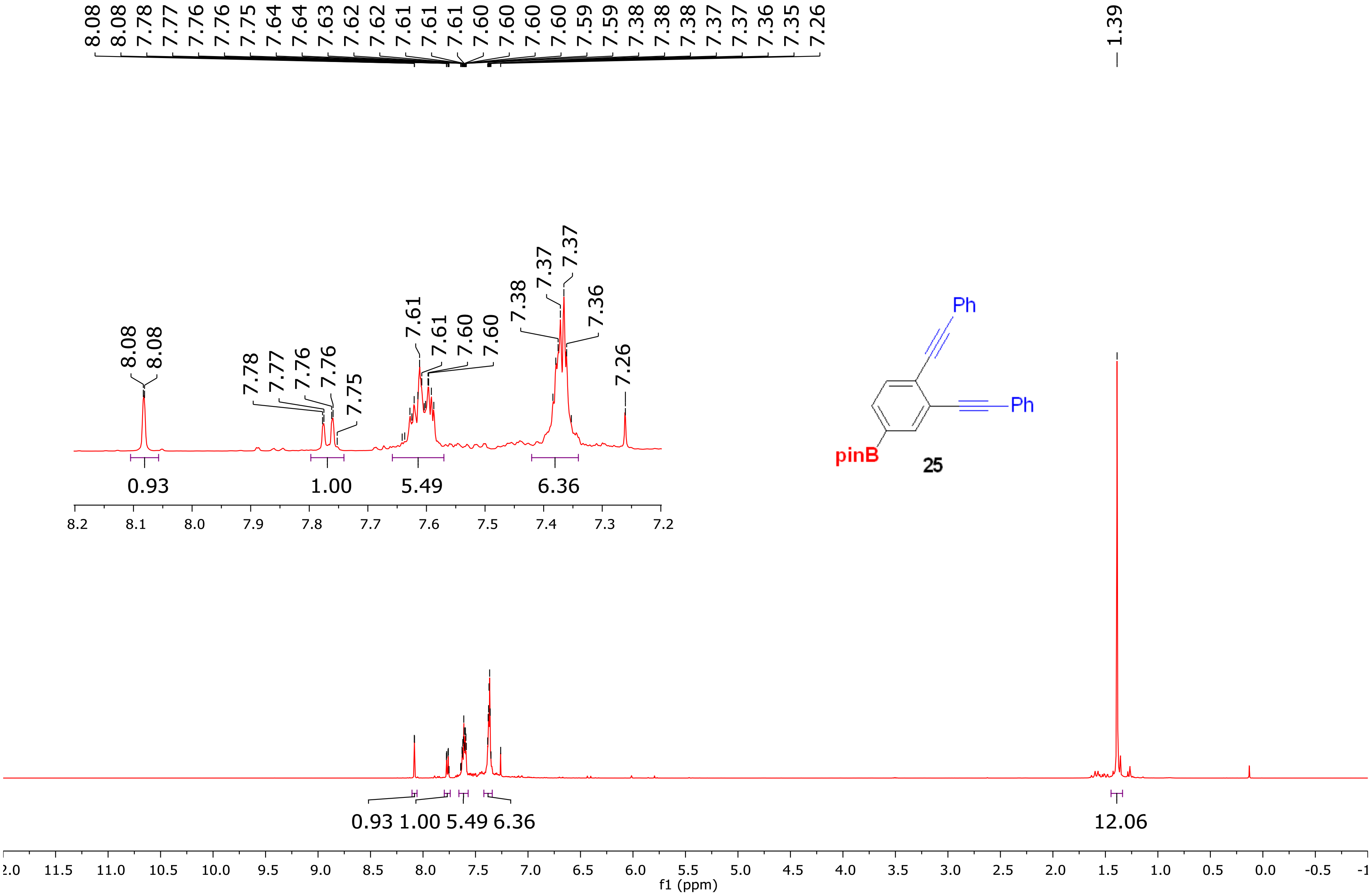

Entry 14: ^{13}C NMR of 25 (CDCl$_3$, 126 MHz)

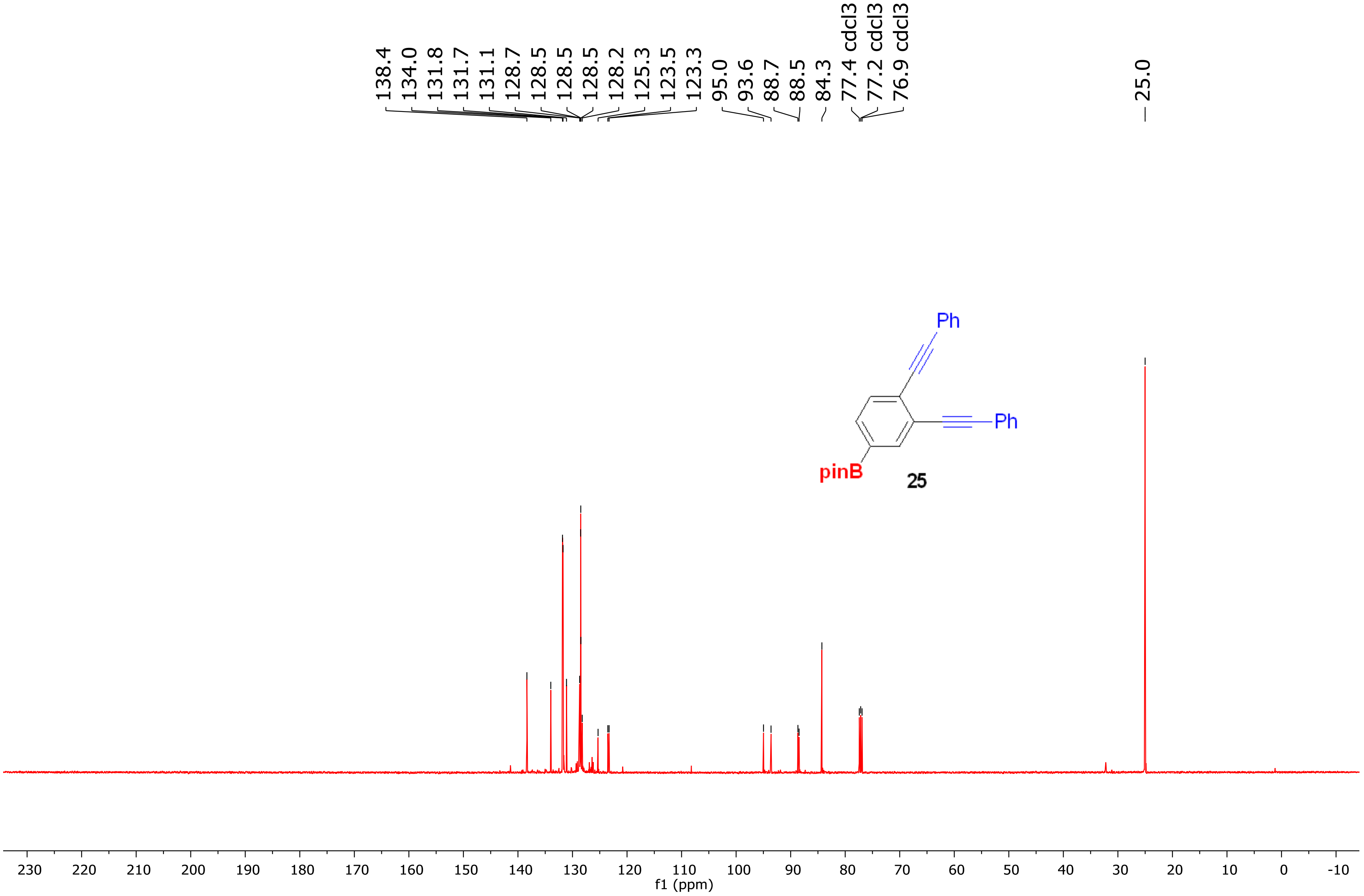

Entry 14: ^{11}B NMR of 25 ($CDCl_3$, 160 MHz)

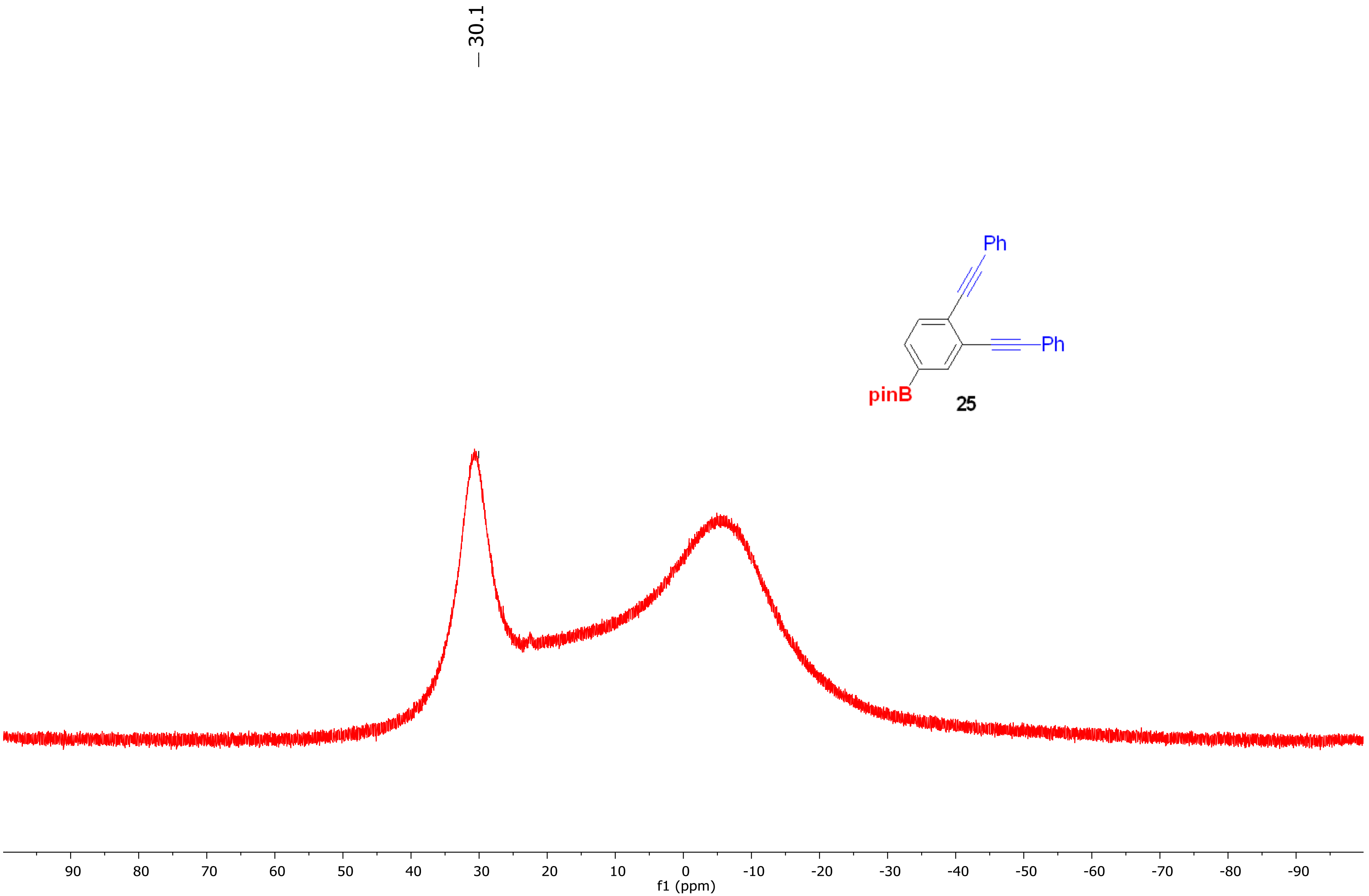

Supplement: Supplementary file 1 [file molecules-25-01754-s001.pdf]
